# Envy's pathology: Historical contexts

**DOI:** 10.12688/wellcomeopenres.10415.2

**Published:** 2017-05-23

**Authors:** Lina Minou

**Affiliations:** 1 Independent Researcher, Nottingham, UK

**Keywords:** envy, physiology, melancholy, corrosion, cancer, early-modern, eighteenth century

## Abstract

This article is concerned with the physicality of envy primarily in early –modern, but also in eighteenth-century health contexts. The discussion brings together descriptions of the effects of envy on the body of the envier, mainly from works of physiology and health preservation, but also from literary and spiritual writings. These depictions of envy are studied beyond their symbolism and with a view to establish whether they are meaningful according to the medical theories of the time in which they occur. The discussion begins by acknowledging the status of envy as a ‘disease’ and looks to the specific ways in which the discourse of envy conveys this sense. I find that in the early modern discourse envy is always pathological, that is, it is experienced as disease and signifies disease in general and several diseases in particular. Moreover, envy is uniquely placed to convey pathology on account of its being connected to inherently pathogenic elements of the humoural theory. Specifically, envy is physiologically connected to melancholy, and the way it is presented comes close to attributes assigned to black bile. In addition, envy realizes pathology, the occurrence of disease in the body, by impairing the vital process of digestion and thus depriving the person from proper nourishment and sustenance. The analysis further considers how this impairment of the body fits with the physiological manifestation of envy as ‘corrosion’ and ‘consumption’. Finding commonalities with other maladies mediated by these physiological signs the article concludes by considering the function of pathology in the conception of early modern envy.

## Introduction

 The early modern period defined envy, in keeping with classical tradition, as ‘sorrow for other men’s good’. ^1^Formal definitions, however, fail to convey the suggestive power of this emotion to the early modern mind. Envy conveyed a horrifying image of debility and physical torment that was most akin to disease. For instance, Robert Burton in his famous textbook on melancholy followed on this traditional definition of envy by referring to the envious as ‘a living anatomy’, ‘a skeleton’, ‘a pale and leane carcasse’. ^2^Indeed, the most important information on the early modern perception of envy derives not from the way it is defined, but from the richness of the language and the powerful images associated with it. Early-modern representations of envy, rooted in the classical and medieval traditions, presented this as a passion of an extremely pernicious nature and the most emphatic way in which they conveyed this message was by references to physiology and, as this paper will argue, pathology.

 The most enduring, literary, image of envy came from Ovid’s *Metamorphoses* where the personification of envy is a grotesque figure with ‘sallow cheeks’, ‘shrunk body’, ‘decayed teeth’ and venom-coated tongue’ ^3^. Middle English versions of envy’s image also follow on that vein of bodily decay. The Middle English allegorical poem of *Piers Plowman* by William Langland presents envy as ‘pale’, with ‘lean cheeks’, resembling ‘a leek that had lain long in the sun’ and having the ‘palsy’ and a ‘body blown up for anger’ ^4^. In the sixteenth-century, the poet John Skelton gave a powerful description of envy, rendered all the more emphatic due to his characteristic style of short, haphazardly rhymed lines. Skelton’s envy has ‘leathery eyes’, ‘dry cheeks’, ‘wan visage’, and ‘creaking bones’ ^5^. The figure is also ‘lean as a rake’, has ‘rusty gums’, a ‘bitter heart’ and ‘liver and lung wrung with anger’ ^6^. Spenser’s envy in The Fairie Queene (1590) is presented as chewing on a ‘venomous toade’ with ‘cankered teeth’ ^7^ Many other examples could be produced. In all of them, the physiological, that which refers to the body, always forms part of the representation of envy.

The connection with corporeality and connotations of disease have always been part of the discussion of envy. Traditionally, envy’s association with the evil eye, rooted in the sense of its Latin origin of *invidere* – to regard with ill will – elicited notions of harm akin to the spreading of disease. Proverbial advice to not share bread with the envious or to protect oneself from the envious person’s envenomed breath spoke to the contagion paradigm under which the influence of this emotion in society was perceived. Envy had the capacity to cause strife within society and also to directly cause harm to individuals who were the objects of the evil eye. An array of preservation and protective practices, observed across many cultures and historical periods, attests to the pervasiveness of this concept ^8^. In addition to being viewed as a disease within society, envy was described in terms of disease for the person experiencing it. Defined as pain or distress at the sight of others’ prosperity, envy necessarily incorporates the element of suffering. Although this aspect of the experience of envy can be said to be in common with other negative emotions, discussions of envy usually point to this suffering as especially heightened and harmful to the wellbeing of the individual. Bridget Balint traces the origin of the emphasis on the harm caused by envy to the envier himself to the religious discourse of the Middle Ages when biblical commentators allowed for a novel perspective on classical notions of envy. As she notes, ‘when the Fathers write in detail about the term and its meaning, they seem less concerned about the damage the envier might do than about the spiritual health of the envious person’ ^9^. Religious discourse, and more specifically the discourse of the seven deadly sins, enhanced this notion of envy as disease and the envious person as a sufferer. Firstly, the discourse of vices favours the metaphor of spiritual ‘health’ to which envy is presented as detrimental. Secondly, the scheme of the seven deadly sins, or vices, developed into a genre with a series of well-defined conventions among which the medicine metaphor was prominent ^10^. In turn, this created and established links of envy – one of the deadly vices – with specific diseases, mainly leprosy, jaundice, and fever ^11^.

The present article also focuses on the detrimental effect that envy has on the envier, but it does so from a strictly physiological perspective. That is, it is concerned with the effects of envy on the body of the person experiencing it as these are recorded in discussions of envy in early-modern and eighteenth-century context. The connotations of disease and bodily suffering that are associated with envy are not lost to scholars. Critics recognize this aspect of envy and discuss its significance. However, in most discussions the physiological traits of envy complement the analysis rather than being its main focus. For instance, F.N.M. Diekstra, in an article on medieval envy, points to the rhetorical power of these images of bodily decay. The rotting body, the thinness, the paleness, the fever, the sickly complexion, all converge towards one important message: To denounce envy is not only the spiritually right thing to do, but also the rational thing to do, as one is spared the physical suffering it entails. Diekstra convincingly argues that the potent body-related imagery, which accompanies the description of envy, is one of the most powerful weapons in the rhetorical arsenal of medieval moralists ^12^. Lynn Meskill, discussing envy in an early modern context, points to envy’s physiological traits and to the contagion paradigm that follows this emotion from ancient and medieval times, in order to explain envy’s perception as a disease that requires a potent antidote. In turn, this antidote is found in Ben Jonson’s drama ^13^. Literary critics who discuss the treatment of envy in poetry, such as Milton’s envy in *Paradise Lost*, also take into account the emphasis on the bodily decay to show this emotion’s base nature ^14^. Such discussions offer important insights into how this emotion was perceived historically. However, they are not primarily concerned with physiology, and for this reason treat the physicality of envy largely as symbolic and as purposefully hyperbolic. This approach is aided by the fact that the physiological characteristics attributed to envy are ostensibly overwrought. It is also common in analyses, with some exceptions, to regard physiological aspects in earlier works under the prism of medical metaphor. This is especially the case when the concept under study relates to the concept of sin or to the passions. In this sense, studies tend to focus on the extravagant and the abstract in descriptions of envy, which confirm the trope of disease as metaphor without necessarily accounting for the pragmatic basis of these descriptions My aim is to consider the physiological elements in descriptions of envy in order to establish whether they are meaningful according to the relevant medical paradigms of the time in which they appear; mainly the period from the 1500s to the late 1700s. In seeking to explore the physicality of envy in the past this article is aligned to research in the field of the history of emotions and health.

In recent years there has been a renewed interest in the research field of the history of emotions. New approaches to the history of emotions have allowed us to expand both on the paradigms with which we analyse emotional experiences of the past, and also on the kinds of sources available to the historian. They have also resulted in a surge of research inquiry in related fields, such as social and cultural history ^15^. A similarly revived interest is noted in the field of emotions in health contexts. Specifically to the period of study here, the volume on *Emotions and Health 1200–1700* edited by Elena Carrera, where she also contributes a significant study on the physiology of anger, and the work of Fay Bound-Alberti, are significant. The work brought together by Carrera paints a complex picture of the experience and representation of emotion in the medical discourse of the medieval and early modern periods. Overall, it showcases the affinities and tensions among the many discourses, cultural, philosophical and literary, that overlap with the medical view, painting a complex image of the connections between body, soul, and mind in the period. It also very usefully points to the idea of moderation in emotional expression as a contributing factor to health and wellbeing ^16^. Bound Alberti’s edited volume is concerned with a later period, from the late seventeenth century onwards, when new discoveries in science helped shape a different framework of the workings of the body, firmly separating the corporeal and the non-corporeal, and articulating new ways of explaining the effects of emotions on the body ^17^.

In general, recent scholarship on the history of emotions has done a lot to revise both our reductionist view of emotions in the past in general, and also to revise the solely negative view of specific emotions, such as anger. However, envy remains under-represented in research. There is no work of the same scope on envy as there is, for instance, on anger by Rosenwein ^18^, or on anger and physiology by Carrera ^19^. The present work aims to contribute research to one of its most defining aspects; its physicality. I will look to discussions of envy within a health context in order to account for the unanimous condemnation of this emotion as dangerous to one’s health and wellbeing. I will also take into consideration the language used to describe envy in the period, both literal and metaphorical. I will seek the literal basis of metaphorical and symbolic language, as my research suggests that hyperbolic images often have a basis on actual physiological principles. In this way, my work comes close to recent research by Virginia Langum on medicine and the seven deadly sins. Similarly to Langum, I also find the foundation of envy’s physiology in the melancholy humour and in anger. My work covers a later period, though, and also entails a consideration of the function of the pathological discourse accompanying envy. The terms ‘pathology’ and ‘pathological’ are used here to denote deviation from a healthy condition and engenderment of disease. They also denote deviation from the norm: that which is considered as unusual and extraordinary, with adverse effects on health.

## Methods and sources

To explore the physiology of envy in the past, I intend to focus on sources that are associated with medicine and health. Primarily this suggests texts that fulfil two criteria: firstly that envy is being discussed in terms of its effects on the body and, secondly, that there is a clear objective towards the preservation of health or cure. The sources discussed here were identified using advanced, subject, and also proximity search combinations in the databases *Early English Books Online* (EEBO) and *Eighteenth Century Collections Online* (ECCO). The subject searches enabled me to focus on the texts that relate specifically to health, whereas proximity searches allowed for a more extensive overview of the language associated with envy and also the organs of the body related to it. All the texts referred to here can be accessed electronically through these databases and can also be consulted physically at the British Library via the English Short Title Catalogue (ESTC). The manuscripts were located through the specific database compiled by Voigts and Kurtz on *Scientific and Medical Writings in Old and Middle English* (eVK). More details on the ways these works can be accessed will be provided in the course of this discussion and in the reference section.

In keeping with the objective of this paper, the primary sources consulted are mainly health manuals. They represent a long-standing tradition of writing on the principles of prevention rather than cure. Their objective is to educate readers in the best practices and habits that will help them preserve their health and prolong their life. These texts, called regimens of health, are an integral part of the Galenic view of medicine, an ideology that, in its many forms both sustained and renewed, largely informs the medical knowledge and practice of the period under consideration ^20^. These manuals profess their usefulness and their accessibility to a wide and, most importantly, lay audience. The fact that they were written in English rather than Latin is a testament to their intended readership. However, it is difficult to ascertain the degree to which their advice reached all parts of society. Their use presupposed a certain level of specialized knowledge and, despite the fact that they devote sections to explaining the medical ideology that informs their content, the kind of language they use is often complex, filled with obtuse terms, and allusions to classical literary works and authors. Although these sources have limitations with regard to the voices they represent, their purpose and content makes them indispensable in appreciating the effects of envy on the body as they were understood in the period in question.

Another set of sources for this paper derives from religious writing. Although not obviously relevant to the present reader, works such as devotional aids, sermons, and morality tracts are essential in accessing information about emotional experiences of the past. In the early modern period the immaterial is intrinsically linked to the corporeal through the indissoluble connection between body, mind and soul. The management of the passions for the sake of health is not strictly the realm of the medical writer. Approaches on the discipline of the passions were offered from medical, religious, and philosophical perspectives ^21^. The boundaries between these perspectives were often blurred, as were their discourses. For instance, there are works consulted here that combine moral and bodily hygiene, such as John Harris’s *Divine Physician* (1676), which blends medical knowledge and moral precepts. Alternatively, there are works with a clearly moral objective, such as John Scott’s *Christian Life* (1696) that borrows from the bodily experience of envy in order to evidence its dangerousness for the soul. Sources of religious nature can be said to be more inclusive as their content could circulate in both published and oral forms. However, oral dissemination does not necessarily imply a wide audience. For instance, Scott’s text was compiled for publication from material derived from his university lectures.

These sources, then, cannot recreate an early modern representation of envy that would be meaningful across all social strata. There are obvious limitations of representative voices in them. However, their subject matter allows us to access a part of the physicality of envy in the past. In its turn, this reconstructed aspect of early modern envy is also subject to limitations. The present study can only hope to understand envy within the medical context of early modern England in the period from the late sixteenth to the late eighteenth centuries. The insistence with the physicality of envy is pervasive enough to be recognized in many cultures and periods, but the effects of envy on the envier are predicated upon certain notions regarding the workings of the body that are not immutable. This discussion, therefore, pertains to the effects of envy as these were circumscribed by the specificities of a medical discourse that operates in England of this period and which is synthesized by the fundamentals of Galenism, the emergence of opposing ideologies to it, and an inherent reciprocity between the medical and the cultural register. This latter aspect suggests a tendency to recognize commonalities between the organic and the socio-political body and thus to create rhetorical affinities between organic and social disorder and pathology. Under these conditions, envy acquires significant power to denote social disharmony and disorder, a capacity which is reflected on its representation as pathological. What is more, under these geographical and cultural conditions envy becomes an emotion of particular interest and concern ^22^. The questioning of established hierarchies that progressively takes place in England over this period, culminating in the political upheaval of the seventeenth century and the experimentation with parliamentary power, as well as its aftermath, places focus on envy as a dangerous emotion that is rooted in discontent. In turn, this concern with the social significance of envy is pertinent on the physiological discourse discussed here. In brief, the conception of envy as pathological presented here may not apply to a broader European context, but is particularly telling within the context of early-modern English medicine and society. The analysis first pauses to consider the principles that inform the early-modern framework of emotions and health. This section offers essential information on early-modern physiology so that the position of envy within this framework of health and disease can be appreciated. It also explains the necessity for diversity in the kinds of sources use for the study of envy in this context.

## Background on emotion and health

With the exception of a small number of manuscripts, the texts used here are usually print sources drawing, directly or indirectly, from the principles of the Hippocratic-Galenic tradition ^23^. This means that they subscribe to a humoural interpretation of illness and cure. Under the humoural paradigm, the aetiology of disease could be found in some kind of disturbance in the systemic balance of the four recognised humours in the body: blood, yellow bile, black bile and phlegm. Each of these humours corresponded to particular qualities that they themselves invoked cosmic elements. Blood was moist and hot, like air, yellow bile was hot and dry, like fire, black bile was cold and dry, like earth, and phlegm was cold and moist, like water. In every individual, one of these humours was perceived as being predominate ascribing to that person a particular ‘complexion’. Complexion was a person’s humoural type and influenced their personality traits and physical appearance. A predominance of yellow bile, for instance, resulted in the choleric type. Knowing one’s humoural type was crucial both for prevention and cure, as each of them made one susceptible to different kinds of diseases. In humouralism, a state of absolute health was never in fact attained. What was sought, instead, was a state of equilibrium, a best possible balance of the humours within the individual. Although this was the ideal, the individual was prone to different misbalanced states, in which any one humour would prevail in an abnormal way, with adverse effects on health ^24^.

Many factors could influence the state of the humours in the body. For this reason, in Galenism prevention was as important as treatment. The most effective method of preventing illness was practicing moderation, especially with regard to the six *non-naturals*: 1. Air; 2. Sleep and waking; 3. Food and drink; 4. Rest and exercise; 5. Excretion and retention; and 6. The passions ^25^. Thus, preserving health entailed observing such rules as avoiding exhaustion, overeating, and indulgence in excessive passions. The passions were in need of moderation, since the complex unity of body, soul and mind underlying this tradition means they could act upon the spirits and humours and influence the humoural balance. Conversely, they could also be affected by it ^26^. All passions were in need of moderation, both ‘positive’ and ‘negative’ ones. Joy, for example, was generally perceived as conducive to health, but sudden or excessive joy could have adverse effects. On the other hand, moderation could mitigate the effects of negative, harmful passions, such as anger, and even render them useful. For instance, Elena Carrera’s work has shown how moderated anger could be beneficial to health, or even therapeutic. Humouralism abided to the tenet of curing by contraries and for this reason, Carrera explains, the heat induced by anger could countervail the effects of coldness in the body ^27^. Envy exists in no such positive state. This is because it is difficult to imagine gradation in envy and thus difficult to moderate it. Indeed, the positive counterpart to envy is a different emotional state: emulation. Although the distinction between envy and emulation appears in philosophical discourse, with regard to physiology the discussion is precluded to envy. The term ‘envy’ is the one being discussed for its effects on the body which are universally acknowledged as deleterious. The sources used here all use the term ‘envy’ and they imply malicious envy. They either focus on this term solely, or they discuss it within groups of certain other emotional experiences such as hatred, malice, jealousy and ‘revenge’; treated as a passion as in the desire for revenge. All of the above denote particularly opprobrious emotional experiences with no moderate state.

The majority of the sources studied here belong to the genre of hygiene and the way the effects of the passions on the body are incorporated within the advice contained therein. However, as is evident from the literary descriptions of envy cited above, other kinds of sources, not obviously relevant to medical history, are also interesting in terms of what they reveal about envy and health. Such flexibility in the inclusion of sources is necessary, due to the paradoxical nature of the discussion of envy. Finding evidence of the effects of envy on health is not as straightforward as consulting texts that traditionally address the subject of the effects of the passions on health. Envy is an emotion that is always described with reference to physiology, as noted above, but it is not an ‘interesting’ emotion in terms of physiology. I will clarify this phrase below.

Medieval and early modern medical writers explained the physical modifications caused by the passions through the concepts of natural heat and the movement of the spirits ^28^. The heart was the epicentre of the process. In the words of Pedro Gil Sotres: ‘the emotional dynamic was triggered by the introduction of heat and spirits into the heart or by their outflow from the heart’ ^29^. This centripetal or centrifugal movement, as well as the quickness or slowness of this movement, is an integral part of the medical explanation of the effects of the passions. It is evident that under this theoretical system certain emotions have greater explanatory power. Anger, for instance, is fit for illustrating both the direction and intensity of movement: a quick displacement of the spirits and heat away from the heart. Conversely, fear is equally potent in illustrating the opposite effect, while joy was the obverse of anger in instigating a centrifugal but slow movement of spirits and heat. Other passions do not share the same capacity. For this reason, the list of emotions that interest the medical writer is shorter than the list of emotions that interest the philosopher ^30^. Envy occupies the space between these two concerns: it is one of the most interesting emotions in philosophical and spiritual terms but it does not appear in the list of emotions that illustrate the effects of the passions on the body. For this reason, the data that inform an analysis of envy from the point of view of its physiology are necessarily fragmentary and derive from a multiplicity of sources. In this article, selected paradigms from spiritual and literary writing will also be included as long as they help illustrate a specific aspect of the physiology of envy.

The article begins by looking at the language associated with envy with a view to establish the main qualities of this emotion that underpin the discourse of its dangerousness. It proceeds to position envy within the humoural framework and to explain its aptness in suggesting pathology. The following section discusses how the diseased state is not only threatened, but realized in the envier’s body. Finally, the article concludes with a consideration of the main physiological signs inscribed on the body of the envier and analyses their significance.

## Envy and language

Most of the physiological information on envy derives from simile and metaphor. Envy was likened to a series of specific diseases, many of them recognizably grave. Envy is like ‘leprosy’, the ‘plague’, most often it is a ‘consumption’, a ‘worm’, a ‘canker’ on the flesh, an ulcer, like ‘cancer’, and also like ‘a hectic fever’. On the one hand this impressive array of ailments speaks to the power of physiological metaphor itself rather than offering evidence for the physiological composition of envy. These are all diseases that captivate the popular imagination, intelligible through their effects and the impact on the population, without reference to medical authority. On the other hand, they do bear significance for the physicality of envy. A disease like the plague may be used as a metonymy for disease in general. By contrast, hectic fever had different connotations. It was a very specific pathological condition that was the result of loss of radical moisture. According to the principles of Galenism, radical moisture, with which humans are endowed from conception, feeds the innate heat of which life consists. Natural death could be understood as the dissipation of this natural heat. Hectic fever is the intensification of this natural process. As such it suggests and also threatens fatality ^31^. This tendency to view envy as potentially fatal is usual. Indicatively, a middle English poem on envy, illustrating St Augustine’s definition of this passion, uses four consecutive similes to describe the action of envy closing with the line ‘as death to life’ ^32^. This tendency exists more or less in all advice against envy helped by language that stigmatises it in a very specific manner.

Although there is great variety in the language associated with envy, most metaphors converge on the sense of disease in general and on ‘corrosion’ in particular. Proverbially, envy is ‘the rottenness of the bones’. Other common phrases include ‘as rust to the iron’, and the ‘worm that breeds in the timber’. John Harris (1666–1719), writing in his *Divine Physician* (1676), used these phrases to emphasize this aspect of envy:

Envie to the heart is like rust to the iron, or blasting of the corn, like the vultures eating up continually the heart of Prometheus, or the foolish bee that loseth the life with the sting: it burneth the heart, and wasteth the body, and is like the worm that breedeth in the timber and consumeth it ^33^.

The sense is one of slow, but sure, inward erosion of the body. In non-metaphorical discourse, envy is also frequently termed as ‘corrosive’ or a ‘corroding’ passion. Common verbs associated with envy connote erosion and attrition. They include terms such as: ‘waste’, ‘wither’, ‘starve’, ‘pine away’, ‘consume’, ‘burn’ and ‘eat’. Most of these verbs converge on a sense of ‘eating away’. Jonathan Blagrave (1652–1698) discussed the nature of envy in his 1693 sermon on the subject using a series of verbs that denote erosion. ‘Envy’, he noted, ‘pines [a man] away, it wastes his flesh, consumes his bones, eats his very heart’ ^34^. In general, a sense of the enfeeblement of the body always follows this emotion. ‘Envie’, Harris noted in 1676, ‘is the worst of all passions; and feedeth upon the spirits and they again upon the body’ ^35^. In 1600 in his work concerned with practical advice on health, William Vaughan (1575–1641) noted the physical aspect of envy: ‘it maketh a man to looke leane, swart, hollow eyed, and sicklie’ ^36^.

The language of envy illustrates the fact that this emotion is extensively connected to pathology. Envy is ‘disease’ in its generic form, bestowing on the individual experiencing it the look of infirmity. Moreover, it has the capacity to signify different diseases, greatly dangerous to the sufferer. More than that, it is connected to fatality and incurability. The physiological attributes of envy are at once generic and specific. Leanness and wasting of the body can connote sickness in general, but along with dryness can point to a specific physiological composition: that of sorrow and the melancholic type. However, sorrow is not adequate to describe the experience of envy. It is considered a composite passion and also includes anger. Envy’s materiality involves both of these emotions, but to different degrees. Anger, as the present analysis will show, is present in the envious body, but does not affect it to the same extent. Dividing envy in its core elements is useful because it leads to specific and recognizable physiological effects. Anger and sorrow are paradigmatic passions in the list of the medical writer. They are also contrasting in their physiological effects and this bears significance for the physiological make-up of envy. This will be made clearer in subsequent parts of this article. Before going on to present this aspect of envy’s materiality, the following section will firstly address the physicality of anger within the emotional experience of envy.

## Envy and anger

Envy and anger are traditionally paired together in the moral and literary discourses. In Spenser’s *Fairie Queene* (1590), for example, envy is presented riding on a wolf and wrath follows. Moral and pastoral texts can argue the harmful nature of these emotions, as they contradict notions of moral behaviour towards others. Both envy and anger threaten harm to others, often without moral justification. There is evidence to suggest that they are paired together in health writing as well. There are manuscript sources that pair these emotions together strictly for reasons of health preservation. Three manuscripts in the Sloane collection, held at the British library, each contain a folio that repeats the same advice against anger and envy. The sentence is always located in the opening section of a text that offers dietetic and other advice on health and reads thus: ‘If thou will keep long health/then keep this rule/that is to say flee anger wrath and envy/give thee unto mirth and mean travail’ ^37^.

The specific manuscripts all belong to the scientific or medical tradition. Their contents are diverse, but all the material contained relate to proto-science or medicine. Their titles also attest to this. In more detail, they are: MS Sloane 213 ‘Medicine Charms and Receipts’; MS Sloane 1609, ‘The Wise Book of Astronomy’; and MS Sloane 2270, ‘Eamys, Book of Medicines’ ^38^. The quote above is directly taken from MS Sloane 2270. All other texts feature the same sentence with some variations. The Sloane 2270 text begins with the incipit above and goes on to give specific advice on the preservation of health: avoiding sweat, especially in the hot summer days; the ways to eat meat; proper eating in general, including avoiding of overeating; and advice on when and how much to sleep. Other subjects that occur within this text, and also in the other compilations referred to here, are astronomy, geometry, lucky and unlucky days, physiognomy, complexions and humours, and the positioning of the stars, as this is relevant to the taking of certain medicines.

There are enough similarities in these manuscripts to suggest that there is a common origin for the advice on anger and envy. However, the advice contained in this specific part of the texts – as well as in these compilations in general – suggests that the information presented is common knowledge. Tellingly, the text that precedes the incipit on preserving health in the 2270 Sloane manuscript is one that explains the consequences of ‘thunder’ occurring in each month. This kind of advice is practical and eminently useful and usable. In the whole text introduced by the particular incipit, no medical authority is cited and bodily function is not explained. The advice provided needs no reference to authority. Some rules, for example, include: ‘flee strong drinke [...] and burning meate’; fast not long in the morning ne fast too late in the evening’; ‘sleep well in the weare of the night and be early up in the morning’ ^39^. It is easy to remember and disseminate these rules. They are simply phrased, and due to the fact that the text states rather than explains these rules for health, they acquire a kind of mnemonic quality. The significance of these findings lies with the nature of the texts. These manuscripts single out anger and envy among the passions as deleterious to one’s health for reasons that are not connected to sinful behaviour and moral decay. The practical advice of these texts and the inclusion of anger and envy within that context show that these emotions are commonly accepted and understood as injurious by nature. On the other hand, the fact that advice against these passions is offered within a context of dietetic advice on health may also have its meaning. There is evidence to suggest that envy is shown to affect the viscera and to be counter-indicated in diseases of the abdomen and the intestinal tract. For instance, Jean Goeurot’s *Regiment of Life* (1550) specified ‘yre, envye and melancholye’ as passions to be avoided in diseases of the bowels, especially colic ^40^.

Crucially, anger is also part of envy. Virginia Langum finds that medieval sources merge physiological elements of anger with envy in the body. For example, envy is typically associated with jaundice, and this ailment can be connected to various kinds of choler; the corresponding humour to anger. Most significantly, anger is manifested in the envious body in a particular kind of swelling that is attributed to it. Notably, Langland’s personified envy, described above, has a body ‘blown with anger’. Langum distinguishes between two kinds of swelling in the envious body: one that is visible, which is associated with anger and the movement of vital spirits away from the heart; and one that is internal, which is connected to envy and the withdrawal of spirits towards the heart ^41^. This is a useful distinction. In the early modern period references to swelling by envy persist in phrases, such as ‘puffed with envy’, an envious heart being ‘puffed with anger’, and ‘swell with envy’. These verbs are also associated with pride, and often there is a double construct, as in ‘swell with pride and envy’. Most of these cases use the notion of swelling as metaphor. However, on occasion, the metaphor works by threatening the physical limits and workings of the body. A political text of 1658, for instance, warned that an envious heart is ‘like a tympany which swells up the man until he burst asunder’ ^42^.

 The physical experience of anger also involves the rise of heat in the body. The heat of anger, though, seems to be rendered ineffectual as far as the physicality of envy is concerned. As a text of 1651 explains, anger does not affect the materiality of envy because the heat of choler is negated by the presence of contrasting qualities in envy:

Envie is by accident of a cold and dry nature, having a shrinking quality, like unto that of Fear and Sorrow; for although Wrath, of which it is composed, be hot and fiery, yet being turned into Hatred, it loseth its natural heat, becomes cold by accident, as the humor of the yellow Choler, which is hot, being burned, changeth its nature, and is turned into a cold melancholy humor ^43^.

The excerpt above, explaining the physicality of envy by analogy, effectively states that anger is only nominally part of envy and does not affect its materiality to a great extent. However, this omission is not without its consequences. The heat of anger can be a positively imbued physiological element. As has been noted above, it can be useful and even therapeutic in some cases. More than that, as the element of heat becomes negated, the constitution of envy cannot be mitigated. It thus remains primarily expressed through coldness and dryness, both qualities antithetical to the very essence of life posited by Galenism.

## Envy and melancholy

It becomes evident, then, that the physicality of envy is primarily connected to sorrow. From a physiological perspective, envy is a branch of the melancholic complexion. Texts of regimen categorise envy under the melancholic type. In 1564 Philip Moore published *The Hope of Health*, a text on the preservation of health that aimed to disseminate knowledge of the qualities of medicinal herbs among the poor. As is usual with this type of medical writing, information on the humoural composition of the body is included as the basis of physiological and, mainly, therapeutic understanding. In the relevant section of Moore’s book the melancholic body is recognized by a list of signs. Among these signs are ‘coldness and dryness’, ‘leanesse and roughness of the whole body’, ‘blacknesse or swartnesse of the face and skinne’, and also ‘envie’ ^44^. In Goeurot’s *Regimen*, quoted above, envy is also assigned to the melancholic ^45^.

The idea that a melancholic was physiologically inclined to be envious also had currency in the moral discourse. The fourteenth-century *The Book of Vices and Virtues*, itself a translation of a significant medieval moral work, observed that each person is tempted according to their individual weaknesses. Thus, the devil leads one to temptation by taking advantage of each person’s physiological make-up and the fact that the predominance of a specific humour inclines one to certain sinful emotions and behaviours. Accordingly, he incites the choleric to wrath and the melancholic to envy ^46^. As the prominent writer of theological and devotional literature Lancelot Andrewes (1555–1626) explained, ‘for the melancholy [the devil] laies baites of envy: and so for every one according to their natural inclinations and humours, such baites as may entice them soonest’ ^47^. In 1576 Levinus Lemnius reiterated the notion in his *Touchstone of Complexions* ^48^. Lemnius (1505–1568) was an eminent Dutch physician, but his work was well known in England, going through three editions in translation. The work is an amalgam of religion, rules for health and practical remedies, authoritative medical notions, and also arcane notions on the connection of the Holy Spirit to the spirits of the body. The occultism of *The Touchstone* is not an oddity within an early modern medical context. Although the medical paradigm is ostensibly Galenic, early modern medical discourse was characterized by heterogeneity. Philosophical, metaphysical and also religious ideas could be accommodated within this discourse paradoxically without leading to its questioning. In the words of Margaret Healy: ‘Religious, alchemical, Neoplatonic and Paracelsian ideas are all implicated in the rise of an occult discourse in some medical books’ ^49^.

It is this plurality that reiterates the connection of material melancholy (black bile) to envy within a health context. The fact that Lemnius can incorporate religious notions in his medical text articulates and propagates the connection of black bile with envy. A text that would be based solely on medical theory and without an objective towards medicine as practice might not have done so. Although there is evidence of the association of black bile with envy in authoritative texts of the medical tradition ^50^, it is in the health preservation publications that it is sustained. Works such as *The Touchstone* and, generally, published medical works of the time, are not solely deposits of medical knowledge. They are also vehicles for reputation and authorship. In addition, their objective towards medical practice and therapeutics, rather than medical theory, affords them flexibility of interpretation. Writers can posit their own definitions of health, referring to authorities, but also subsuming authority to experience. Nicholas Culpeper (1616–1654), a prolific author of medicine-related works, could maintain in his *English Physitian* (1652) that the benefits of the thistle to health are important, although contemporary medicine found them controversial. He insisted that a ‘decoction of the thistle in wine being drunk, expels superfluous melancholy out of the body’ ^51^. He found this greatly beneficial as excess of melancholy in the body, leads to ‘many evils’ and also to ‘envy’ ^52^. The implication here is significant because it emphasizes the connection between the humour of black bile and inducement to envy. Thus, separating himself from common opinion, Culpeper preserves an association within the medical discourse that was most commonly found in different traditions, mostly moral and pastoral ^53^.

Culpeper was a physician and an astrologer. This branch of medical discourse, still operating in the early-modern period ^54^, offered additional affirmation of the connection between melancholy and envy. In Astrologo-physical discourse, envy and melancholy are ascribed to the influence of the same planet: Saturn. Texts that use astrological information as part of therapeutics repeatedly articulate the link between Saturn and envy. They also commonly ascribe images of social strife and social harm to this planet. These usually follow on the emotions governed by Saturn, such as envy and hatred, and include war, murder, suspicion, and evil thoughts ^55^. Significantly, this association can account for the fact that melancholy and envy share some of their most important signifiers. Famously, Saturn was the planet governing the melancholy disposition, and it correlated to the spleen ^56^. Saturn was associated with bitterness and coldness, due to its distance from the sun, and with dryness since antiquity ^57^. As accounts expanded from the basic qualities governing the god-planets of ancient astrology to include physiognomic and characterological attributes, Saturn acquired the properties that reflected onto the image of the melancholic person. Bitterness, dryness, excessive leanness, despite consuming food, and the ‘black colour’ became some of the characteristics associated with Saturn and the melancholic – and, by implication, with the envious ^58^.

Envy, then, shares a common frame of reference with inherently pathological and pathogenic elements. Coldness and dryness are qualities that threaten the organism’s vital life essences, moisture and heat; melancholy is a condition that suggests a series of bodily and mental states of disorder; Saturn is vested with a malevolent influence both on the person under its influence and also with connotations of greater strife. These factors all speak to the unique place of envy in conveying the pathological. Taking into account the physiological connection of envy to melancholy, it helps not only to elucidate its dangerousness to the individual and to society, but also to better understand its nature as disease to the one experiencing it.

The discussion of envy that is concerned more with the pernicious effects of this emotion to society, places emphasis on the eyes, the malevolent gaze, the noxious breath, or the dangerous touch. In essence, factors that enable infection and contagion. When the focus is on the ways envy is deleterious to the envier, the discussion turns to the withering of the body and mostly to the abdomen. John Scott (1638/9-1695), a spiritual writer, noted in his work on godly devotion and happiness that envy, ‘swells the hypochondries’ ^59^. A verse satire on social ills from 1683 described envy as a ‘melancholy wight’ and ‘a thin-chopt wretch with shrunk-up gut’ ^60^. The references can be understood as linked to the humour of the black bile. The seat of black bile is the spleen and is most commonly associated with diseases of the intestinal tract.

This association between black bile, melancholy, and envy can help substantiate something about the embodied experience of envy in the past, especially once distinctions about the nature of melancholy are taken into account. Medical writers distinguished the humour of black bile, naturally present in the body and positive in small quantities, from the black bile that occurred in the body as the result of excessive heating of yellow choler. This ‘adust’, or burnt, melancholy did not only cause illness but was in itself the outcome of a process of corruption ^61^. They also recognized three types of melancholia each with its own set of related physical symptoms. Only one of them was located in the head, taking hold in the brain, the others took hold, respectively, in the blood and thus affected the whole body and the abdomen. This last type was specifically localized in the hypochondriacal region of the body, in the organs below the ribcage’s lower cartilage and ‘as Erin Sullivan demonstrated’ resulted in complaints about digestion, weak stomachs, and flatulence. In other words, despite our propensity to view melancholy mainly as a condition of emotional distress, and thus of the mind and soul, its experience in the past encompassed an undeniable somatic reality. The recent work of Erin Sullivan on melancholy corrects this misconception by showcasing the significance of ‘organic disorder’ in the medical register of this condition. Sullivan studied doctors’ manuscript casebooks from the sixteenth and seventeenth centuries and meticulously catalogued the symptoms they associate with the medical condition of melancholy. The record that emerges from this endeavour reveals melancholy as connected to bodily dysfunction and more specifically dysfunction that is concentrated in the guts. The most commonly affected organs are intestinal organs, the spleen, the stomach and the uterus, while the most common complaints are digestive problems and abdominal pain. In brief, Sullivan’s research reveals, in her own words, ‘how embodied and, indeed, how intestinal the condition could be’ ^62^.

Sharing a common physiological framework, envy’s somatic experience also involves the same region of the body and, as further analysis will show, its potential to become pathogenic is specifically linked to its effect on the digestive process. However, if, as Sullivan’s work shows, it is possible to separate melancholy from its vast network of associations and focus solely on its physical manifestation, this is not the case with envy. In envy every symptom and sign, although firmly rooted in physiological principles, is stretched to its limits and because of this it becomes suggestive of something greater than the body. If the experience of melancholy involves intense physical discomfort and digestive complaints, the experience of envy, as the following section will show, is associated with digestive disorder of an extreme kind. In envy, it seems, there is always an interplay between the literal and the figural so much so that physiological symptoms become signs with broad semantic and cultural connotations. Indeed, the physicality of envy on the whole can itself act as a signifier of pathology.

Recognizing the association between envy and black bile goes a long way in explaining the physicality of envy. More than that, the nature of black bile can account for this emotion’s particular aptness for the disease metaphor. Black bile was unique among the humours because it was not as fixedly defined as the others were. ‘The concept of black bile’, Rudolph Siegel notes, ‘was based partly on observation, partly on speculation, and unfortunately, too often on conclusion by analogy’ ^63^. With no firm empirical foundation, black bile lent itself easily to the assumption that it was found at the root of a variety of grave diseases, both of body and mind. This was helped by the extensive and pejorative connotations of the term ‘melas’ (black) in its name. This humour was associated with such disparate diseases, including fever, jaundice, leprosy and mental ailments. It was connected to malnutrition and thus to leanness. It was present in ulcers and cancers and in excessive quantities in tumours and wounds. Its presence often connoted incurability. Difficulty of cure was also commonly attributed to envy. Most of the signifiers of black bile are also signifiers of envy. The diseases that converge on the black bile are part of the language of envy. Envy is like a wound and tumour, an ulcer of the mind, and canker of the body. Envy’s corrosive action on the body can also be referred to an integral quality of black bile. Galen explained black bile’s sharpness and acidity (the quality of oxys) as ‘stinging without heat’ ^64^. Envy, then, acquired many of the attributes of this humoural element. What is more, as black bile was vested with an extensive capacity to convey the pathological, so was envy also capable of suggesting disease. Indicatively, when Lemnius wanted, in *Touchstone of Complexions,* to explain to his readers the dry complexion, he encouraged them to bring to mind the ‘physiognomy and shape of envy’, described by Ovid ^65^. He felt that this image best illustrated the swart, grim face and the body of the person toiling under this condition. Lemnius was not referring to the passions in this section. He was describing, in most vivid terms, a pathological state of the body. The dry state, he noted, was ‘repugnant to the laws of nature’ and a threat to the prolongation of life. By illustrating this state through the representation of envy, Lemnius equates the image of envy with the image of pathology.

## Envy and improper nourishment

The previous sections positioned envy within the humoural framework of physiology as closely linked to the melancholic humour and to the qualities of dryness and coldness. This association helps explain this emotion’s unique capacity to suggest pathology. This section will focus on the ways the pathological regarding envy can be realized. A common element in representations of envy is the idea that it hinders the proper digestion of food and prevents or interferes with the proper nourishment of the body. This is an especially dangerous notion in a concept of health as heavily based on diet as Galenism was.

The medical model that is based on Galenic principles and prevails, in various forms, until the late seventeenth-century posits, in essence, a dietetic view of health. Nutrition is paramount to maintaining, and restoring, the healthy state. The importance of diet and food consumption within this medical thinking cannot be overstressed. Under the early modern paradigm, food is not fuel but replenishment of the body’s vital sources. Through digestion and assimilation food becomes blood, humours, flesh, and spirits. The whole physical and mental framework of the body depends upon proper food intake and, most importantly, proper assimilation of food into the body’s frame and function. In brief, the process can be outlined in these essential steps. Food is broken down in the stomach in a process that was imagined as being similar to ‘cooking’. The breaking down of food was achieved by means of the body’s internal heat. The concocted food would then pass into the intestines to be further refined and for the wastes to be evacuated. Crucially, before this stage of evacuation, the juice, which was the product of concoction, would pass into the liver where it was believed to turn into blood. At this stage the unrefined blood contained all the other three humours. At later stages, as blood was distributed around the body, it was drawn to the organs that further refined it into each humour accordingly (e.g. gallbladder-choler, spleen-black bile). Furthermore, the blood that was the result of concoction, first in the stomach and then in the liver, was drawn to the other organs of the body and assimilated to their nature. The blood that was drawn to the heart was turned into the spirits. Hence in this model, all the operative functions, both physical and mental, depended on the process of concoction and proper digestion ^66^. Conversely, improper digestion is considered especially disruptive of health. In the words of Ken Albala:

An improperly digested meal not only causes mild discomfort but is the origin of many diseases. One upset stomach will have resounding effects through every physiological function of the body. One corrupted food product will foul not only the blood and the humors but ultimately the flesh, the spirits, and the mind that is nourished by them. This is not merely a matter of proper nutrition but the foundation of all health [...] ^67^.

In light of the above we can understand more fully the impact of the many representations that depict the figure of envy in the act of eating. This is a common component of literary representations. Ovid’s enduring image presented envy ‘eating viper’s flesh’ ^68^ while Spenser’s envy chews on a ‘venemous toad’ ^69^. In general, envy is presented as eating flesh, specifically eating raw and rotting flesh. At times, the meal of envy is not a physical one. Langland’s envy, for instance, complains of stomach pains and seeks adequate medicine because he is constantly feeding on envy and ill will and these are ‘hard to digest’:

For many years I might not eat as a man ought
For envy and ill will are hard to digest
Is there any sugar or sweet thing to assuage my swelling
Or any diapenidion* that will drive it from my heart
Or any shrift or shame, unless I have my stomach scraped? ^70^

These representations often include references to all the important organs necessary for the function of digestion: Ovid’s envy has ‘black, decayed teeth’ and a tongue coated in venom ^71^, Langland’s envy obsessively bites his lip and has ‘a serpent’s tongue’ ^72^. Spenser’s envy has ‘cankered teeth’, poison running down his jaw and is presented in the act of chewing, not only his horrid food, but also his own entrails ^73^. The grotesque nature of these meals affirms two of the most defining and contradicting characteristics attributed to envy: that it combines baseness with extraordinariness. Envy is a base emotion in the desires it records, but also vested with destructive, supernatural power in its version of the evil eye. Here, the fictional representation of envy suggests a powerful ability to take food that is raw, poisonous, or corrupt, the ingestion of which would kill any figure defined within normal physiological limits. With the exception of Langland’s envy, which suffers the expected physical effects from its fictional meal, all other representations suggest that horrid food is the natural meal for envy. By itself this depiction reveals something of the nature ascribed to envy. As early modern food is directly assimilated to one’s flesh, the implication is that the food that is similar to one’s nature is more easily digested. Envy’s capacity to suffer a meal that would kill another suggests its horrific nature.

In a system of thought that assigns such importance to digestion and assimilation of nourishment into the body, food can also became a powerful pathogen. This is especially the case with raw or partly digested food. As Michael Stolberg observes, when discussing the experience of illness in an early modern context, lay people may not have been fully conversant with the practicalities of concoction, but for them ‘it was sufficient to know that raw food needed to be heated in the body so as to lose its “raw”, impure, and harmful nature’ ^74^. Indeed, the harm of raw and unrefined food is directly connected to corruption of the body’s processes and a cause of ‘swellings, ruptures and innumerable diseases’ ^75^. Accordingly, the imagery of raw, or half-eaten, horrid food that accompanies depictions of envy would have been a clear sign of this emotion as disease.

Eating, specifically improper or unhealthy eating, was also made part of the description of the envious as well as envy in the abstract. In 1616 Thomas Adams (1612–1653), discussed envy as a spiritual disease, but, as was common, used language that reflected the medical knowledge of the time. He said of the envious that he was ‘a man of the worst diet’ ^76^. Moreover, the language associated with envy refers to this concern with improper nourishment in other ways, both direct and indirect. As has been noted in a previous section, the verbs associated with the effects of envy converge on the sense of privation and withering of the body. In addition, envy is shown to specifically affect the organs and process of sustenance. Envy is said to ‘fret the heart’ and ‘to marre digestion’ ^77^. Other texts show envy associated with abnormal swelling, which also interferes with proper nourishment. Envy, John Scott explained, ‘swells the hypochondries, which by drinking up the nourishment of the neighbouring parts, makes the whole body lean and meagre’ ^78^. Scott, a spiritual writer, essentially provides a physiological explanation for the weakened appearance of the envious body.

In general, the two token signs of the envious body, its dryness and leanness, can be attributed to a lack of moisture and a kind of debility, which is the result of improper nourishment. Excessive thinness directly suggests undernourishment. However, moisture, especially in its sense as a humoural quality, is generated in the body by relevant, appropriate nourishment as well. Indeed, the direct and visible effects of this emotion in the body of the envier seem to be connected to the disruption of the vital process of nourishing and sustaining the body. In a ‘tragical anecdote’ aiming to illustrate the ‘baneful effects of envy’, cited in a work of health prolongation, a young lady is shown in a state of dire physical weakness due to envy: ‘her flesh withered away, her appetite decayed, her strength failed, her feet could no longer sustain her tottering emaciated body, and her dissolution seemed at hand’ ^79^. The lady herself admits that chronic envy ‘preying on her vitals’ proved fatal to her. The actual physical cause of her death seems to be undernourishment and under-sustenance of the body.

In essence, it can be said that the physiological discourse complements the established idea of envy as an emotion connected to deprivation. Specifically, envy records the relative deprivation of the subject experiencing it with regard to the envied person and the envied quality ^80^. That is, envy registers a perceived inferiority of the subject experiencing it in relation to the object of envy. As such, it is an emotion that suggests more about the envier than about the object towards which it is directed. What it is more suggestive of is this perceived lack on the part of the envier. In physiology, this is paralleled in the emaciated body of the envious person. The image of the envious body as a malnourished body, deprived from all vital elements of life undoubtedly bears great power for metaphor, but it is also, in the early modern context, a literal, logical consequence of the nature of this emotion. As envy is to begrudge others of what they have or of whom they are, it also does begrudge the body its nourishment and deprives the body of it. Richard Allestree (1621/2-1681), an influential figure in the seventeenth century and bestselling author of devotional and moral literature, made exactly this point when speaking of the dull eye of envy:

'Tis true indeed that discontent and envy shed themselves into the eie, they dwell there in a cloud, the eie flags and is dull, and do's so certainly betray a niggard, envious heart, that we may see it grudges spirits to its own eies, and do's restrain that current that is to feed them with a vigorous life ^81^.

This passage is of great interest both for the physicality of envy and also for the way it reverses the concept of the evil eye. The all-powerful evil eye associated with envy, which can cause harm, is in reality a harmed organ, deprived of the life-giving spirits. In a healthy body, the blood that is the product of concoction leaves the liver through the venous system to follow different directions and carry nourishment to the whole body. The blood that reaches the heart is involved, along with the intake of air, in the production of the life-sustaining vital spirits. Allestree uses this physiological principle to create an image of envy as suppression of vital nourishment. The envious heart functions in the opposite way to that of the healthy heart: instead of supporting, distributing, and sustaining, it deprives the body. In short, envy signifies and works by negation: it negates sustenance and the proper nourishment of the body. It is this aspect of envy that most aptly materializes the pathology threatened by this emotion.

                                                                                          ***

The representation of envy as pathological and its connection to dryness, coldness, and improper nourishment are meaningful mainly under the humoural paradigm, but appears also for the most part of the eighteenth-century. It also seems to be the case that this representation persists even at times when the Galenic theory meets with opposition, as happened for instance, in the latter part of the seventeenth-century with the Helmontians. Helmontians were supporters of the Flemish doctor Jan Baptista van Helmont (1579–1644), himself influenced by the Swiss physician Paracelsus (1493–1541), who rejected much of the established university-taught medical knowledge. They subscribed to a system of medicine founded on principles of chemistry, but also incorporated religious elements. They opposed many key concepts of Galenism and the idea of humoural imbalance as the cause of disease. They were briefly influential in England in the 1660s. Everard Maynwaringe (b.1627/8) was a Helmontian and advocate of chemical medicine. In his work on the prolongation of life, he also characterized envy as ‘disease’ and understood it as ‘enfeeblement’ of the body. He wrote: ‘Revenge, jealousie and envy are the ulcers of the mind, continually lancinating, corroding or inflaming; introducing a secret consumption, wasting the spirits and radical moisture, and infeebling all the faculties’ ^82^. Maynwaringe does not follow the humoural theory, but he primarily assigns dryness to envy. Although this seems incompatible with the medical philosophy he represents, his work, provides, in fact, a good example of the flexibility of the early modern medical sphere. Within his published works envy appears as a subject in those texts that clearly belong to the category of health preservation ^83^.

These texts include sections concerning with the effect of passions on health and illness, as is common with all texts in hygiene. There are separate sections devoted to explaining the effect of the passions on the body and the ways these can engender disease. These sections engage with physiological description in that they attempt to explain the actual ways in which the passions affect the function of the body and how they can lead to disorder within it. In his descriptions he clearly follows chemical principles both in language and content, finding the cause of disease not in a humoural imbalance but in the ‘awakening of morbifick’ substances in the body. What happens when he discusses the passions in general is different from how he presents information on particular passions. When he discusses envy, and the other passions he clusters with it, he omits any reference to physiological explanation – that is, any reference to how exactly envy procures its effects on the body – and instead directly states what envy is and how it affects the body by using recognizable images, phrases, and analogies with well-ingrained semantic and cultural freight. In *The Preservation of Health* (1669) and *The Method* (1683) envy is ‘like a cancer’ and is represented by a wolf that resides in the breast and feeds upon the vitals and the blood, starving the person who harbours it ^84^. As Alanna Skuse has shown in her work, cancer as a disease and the image of the wolf – on its own and as imagery connected to cancer – are part of an established language of illness with significant cultural weight with early modern culture ^85^. When Maynwaringe uses the image of the preying wolf, therefore, he is certain that its suggestive power will convey envy’s diseased and terrifying nature. That is, while Maynwaringe, as a Helmontian, is capable of articulating a scheme of physiology that opposes Galenic explanation of the passions and their effects and also of causes of disease, he also does not resist the power of culturally ingrained images that originated with the medical model he opposes. It is also not random that Maynwaringe chooses the established language and imagery of envy in works that belong to the genre of hygiene. These writings have a clear function in medicine as practice and, thus, the writer has recourse both to the medical and the cultural register in order to communicate information about the body. In short, it can be said that no major change in the conception of envy occurred with the, temporary ^86^, advent of chemical medicine in the late seventeenth century. On the contrary, Maynwaringe’s writings evidence the persistence of the pathological discourse surrounding envy. They furthermore indicate that this discourse shares terms and signs with the language used to describe the experience of other grave maladies in the early modern period. The next section is devoted to exploring these commonalities further,

                                                                                          ***

## Envy’s transformative pathology

As becomes apparent, early modern envy was inscribed on the body mainly through the image of emaciation, in the lean and dry body, and through the action of ‘corrosion’, the constant ‘gnawing’ and ‘eating away’ of the body. Underlying these physiological signs is a tension between what can be observed and what lies hidden within the physical enclosure of the body. Envy was ‘hidden’ – within the mind and also within the body. It was secretive by definition. Understood as ill-will fostered within, it denoted a secret desire for destruction that would not be admitted to. It was an emotion characterized by duplicity and rendered all the more dangerous by its obscurity. It could be apprehended by its effects, the actual harm supposed to be caused by the evil eye for example, but not while it worked on the mind to produce these effects. ‘Envy’, Francis Bacon wrote in his *Essays* works ‘subtily and in the dark’ ^87^. This combination between obscurity, on the one hand, and a great power for disorder, on the other, marks the discourse on envy from both an organic and also social perspective. Form the social point of view it amplified the destructiveness of envy, rendering the emotion unmanageable, as difficult to be rooted out from society as from the body. Bacon concluded the same text, quoted above, by describing the envier as a ‘man [who] soweth tares amongst the wheat by night’ ^88^. The phrase aptly denotes both the envier’s capacity to harm that which is good and socially beneficial (wheat) and also the capacity to do so unobserved. Envy, that is, was both destructive and ‘hidden’ and, for this reason, unstoppable.

The same characteristics are discernible in the organic discourse on envy, that is, the discourse that is concerned with the effects of envy on the body. To the observing eye the body of the envier holds the same secrets as does the mind of the envier. The physical manifestation of envy, the emaciated body, holds no clue as to the cause of this debility. The body actually receives nourishment. There are no natural, observable, causes for its atrophy. Physical causes of atrophy in the period were a lack of nourishment, excessive purging, or a recognizable ‘acute disease’, such as a tumour. When the body receives nourishment but does not actually benefit from it, it toils under ‘consumption’, a medical term that was used to denote unjustified atrophy ^89^. The pathology of envy, then, exhibits certain characteristics that are particularly resonant in an early-modern context. It is hidden or elusive, powerful, difficult to manage to the point of being incurable, and its defining visible manifestation is the leanness of the body. All of these characteristics are present in the medical discourse of other diseases, or diseased states, that were also terrifying to the early modern mind: cancer and ambition-as disease.

Cancer is the most pertinent of these associations. Alanna Skuse’s recent work on this malady unveils the reciprocity between the imaginative conception of disease and its pragmatic experience in the early modern period. Skuse’s study on early-modern conceptions of cancer reveal a disease that is both rooted in humoural physiology – the corruption, or stagnation and ‘gathering’ of black bile in the body – and also mediated through persistent cultural images that center around the notion of it being ‘devouring’ and ‘duplicitous’. In order to come to terms with cancer’s horrifying effects on the body, and its infamous resistance to cure, the medical discourse oscillated between physiological explanation and the use of zoomorphic images, more specifically, the wolf and the worm (canker). These most effectively encapsulated the experience of cancer as a malady that eats the patient from within. The wolf represented the ‘devouring force of cancer’. The canker-worm denoted this malady’s capacity to slowly consume their victim from within while remaining hidden from view. These images also carried considerable semantic, and cultural, freight that spoke to this malady’s defining characteristics; malignancy and duplicity. The crux of Skuse’s analysis is that these images are not only part of the figural aspect of disease but actually mediate the experience of cancer for the early-modern patient. Skuse chooses the term ‘ravenous’ to refer to the nature of cancer, condensing in one word the conception of this disease as parasitic, in addition to being humoural, attacking the patient from within and eating away at their vitality and power ^90^.

Ambition in early modern context was also a condition that straddled the boundaries between imaginative representation and somatic reality. Progressively conceived as a disease, it was rooted in the humour of yellow choler and was connected to heat and dryness ^91^. Thomas Adams in his Diseases of the Soule (1616) explained that the physiological cause of this ‘disease’ was the “abundance of heat drying up moisture” […] done by hot, cholericke, or salt humours engendred in the stomake , or through fevers burning and Ecticke (hectic)’ ^92^. Ambition had both spiritual and corporeal dimensions. The soul was thirsty for honour and the body was thirsty due to the lack of moisture and excess of heat. More than that, this thirst was ‘immoderate’, as Adams called it. It could be satisfied neither materially, by drinking, nor immaterially by acquiring honours. Similarly to envy, ambition was also conceived as perpetual self-torment: ‘a rack’ upon which the ambitious person tortures himself. Spiritual tradition associated ambition with disease, particularly the plague, and noted also its capacity to remain hidden from view. Burton in the *Anatomy of Melancholy*, following established religious rhetoric, named it ‘a canker of the soul’, ‘a hidden plague’ and a ‘secret poison’ ^93^.

Each of these conditions defines its own organic and social disorder, though a varied list of symptoms and images. However, there are significant commonalities. All three of them – cancer, ambition and envy – converge on certain qualities that both represent and mediate their pathology. Their elusiveness, their powerfulness, and their resistance to cure posit them as particularly problematic. More than that, they suggest behaviours and desires contrary to established hierarchies and order. Ambition suggested discontent with one’s place within a hierarchical order while envy could imply the questioning of the division of goods within society ^94^. Cancer, as Skuse explains, was also associated with socially and politically resonant concepts, specifically ‘treachery, treason, and moral failure’ ^95^. All three of them become visible and decipherable only once they have effected considerable damage to the body and, by extension, the social body. Bringing into sharp focus these qualities ‘corrosion’ became an apt way in which to make sense of the terrifying and the invisible. The term ‘corrosive’ itself as a physiological term was defined either as ‘biting’ and fretting’ or ‘gnawing and biting’. The potential for both the physical and the emotional distress is included in the very term. ‘Corrosive matter’ was defined by ‘malignancy’ and always affected the ‘neighbouring parts’ of a wound or an ulcer ^96^. This capacity to spread and affect other healthy parts of the natural and social body denoted the early modern anxiety with the organic and moral degeneration threatened by these maladies, or these emotional states -as- maladies. The description of ‘corrosive’ action along with the relevant imagery of each of these states succeeds in making an obscure and significant threat visible. It also creates a vocabulary for dealing with their consequences. Thomas Adams for instance, suggested ‘vinegar and water sodden together’ for curing the ‘bodily disease’ of excessive ambition ^97^.

However, envy is unique among the diseased states discussed here because it is not only rendered visible but also visibly monstrous. Ambition is clearly a diseased state. The language is ostensibly medical, as is the suggestion of cure. Cancer includes both body references and also animalistic metaphor in its register. The zoomorphic images associated with it can be said to undermine the sufferer’s humanity. However, envy extends the limits of the physiological representing the envier as other than human. This is achieved mainly through a transformation that rests on physiological properties such as strange colour, poisoned and poisonous body members, the combination of human and non-human bodily members –snakes as hair for example – , ingestion of horrid food, and excessive leanness. The most defining physiological signs that envy inscribes on the body not only transform the envier, by undermining their naturalness and humanity, but they also work to weaken the envier’s vitality and power. Overall, envy’s pathological register points to the annihilation of the envier’s body. Terms such as ‘pining’, ‘withering’, ‘wasting’, ‘emaciation’ and ‘atrophy’ suggest the actual diminishment of the physical dimensions of the body. Terms such as the gnawing’ and ‘eating’ of the body register the same desire to erode the body of the envier. In this sense, the action of ‘corrosion’ that accompanies envy is a self-consuming act.

The fact that the discourse of ‘corrosion’ is extended in envy towards the total annihilation of the subject suggests a function for the pathology of this emotion. It seems that the association of envy with the pathological responds to the anxiety over envy’s obscurity. Ambition is felt inwardly, but can be known to others. Its symptoms, Adams says, are ‘words’ ^98^. That is, ambitious persons reveal their desires. They communicate and articulate them. Cancer, on the other hand, is elusive and can be terrifyingly indecipherable. However, it is the doctor’s responsibility, and struggle, to diagnose it, to make it known through making its signs intelligible. Envy’s obscurity differs in that this emotion remains hidden from others but is known to the envier. The anecdote on envy included in the translation of Cornaro’s health manual, cited above, makes this point very clear. In that story there is a division between what is known and observable to all – a lean and progressively debilitated body – and what is known only to the envier herself. The story concludes with a death-bed scene where the young lady gathers her friends around her and confesses to her envy. She reveals that all her life she envied her sisters and that ‘this odious passion’ is the reason for her sickness and untimely demise. This admission does not change the conclusion of the story. It does not act to save or cure the envier. It is not therapeutic in any way. It is an act of revelation that serves to make the story of the envier paradigmatic. Her story now serves as a deterrent from envy. The unambiguous repudiation of envy in the story suggests concern over this emotion’s capacity to remain concealed. It is notable, for instance, that in the anecdote above the young lady’s suffering was unintelligible even to her ‘most intimate’ friends before her revelation.

Envy’s pathology, then, transforms this emotion from well-concealed malevolence that is powerful, into revealed, strikingly visual debility which represents powerlessness. The way this transformation is achieved is pertinent to the two main physiological signs of envy, emaciation and ‘corrosion’. These two terms may converge on the same notion of ‘eating away’ or ‘gnawing’ but they map onto differing connotations. Corrosion represents action, invisibility, potency, and, above all, the capacity to spread. As such it concentrates all the aspects of envy as a passion threatening social disorder. Emaciation on the other hand, represents passivity, visibility, and powerlessness -especially in the sense of visible powerlessness. Most importantly, as a physiological sign of envy it represents the confinement of its effects within the physical enclosure of the envier’s body. By confining the effects of envy to the envier alone pathology figurally transforms this passion from society’s concern into concern for the individual. Envy may work ‘subtily and in the dark’, but it works now to destroy the body that harbours it.

## Conclusions

This article showed how the pathology associated with envy is constructed and mediated according to principles of humoural medicine and the persistence of this pathology in the early modern period even when there is a challenge to the predominant medical theory – as happened for instance with the temporary advent of chemical theories in the seventeenth century. It also further provided commentary on the cultural significance and function of this pathological conception of envy.

The analysis positioned envy within a humoural framework of disease and revealed that in this context it is inherently pathological. That is, it always experienced as disease and engenders disease. Firstly, the nature of this emotion is closely linked to elements that denote deviation from a desired healthy state, namely, black bile and dryness. If the humoural paradigm posits a spectrum of health, rather than an absolute state of it, then black bile and the qualities of dryness and coldness are positioned the furthest away from the ideal condition. Dryness and coldness directly contradict the necessary qualities of life – moisture and heat – while black bile is a controversial humour with few benefits. Secondly, its action on the body interferes with vital processes of both health preservation and also cure ^99^, mainly proper digestion. The connection between envy, black bile, and melancholy in early modern physiology elucidates the organic, physical distress that results from the experience of this emotion, but also emphasizes the fact that the organic cannot be easily untangled from figural constructions of pathology in envy. The physical effects of envy concentrate on the same region of the body as those of melancholy - when the latter is viewed as medical, somatic condition centring on the hypochondries - but they also signify more than distress. As the section on envy and improper nourishment showed, envy does not merely lead to digestive complaints but manifested in the body as a kind of atrophy that is extreme and unnatural and for this reason unsustainable. Indeed, as is reflected in this article, the constant feature in an otherwise highly diverse account of envy’s physicality is the fact that the somatic experience of envy is so toxic that it ultimately devastates the body of the envier. This is echoed in the language associated with envy, so insistent on fatality. It is also epitomized in the overarching imagery of body wasting that represents envy.

In envy body wasting is mediated through two closely connected but separate terms: ‘corrosion’ and ‘emaciation’. The analysis showed that the interplay between the associations of these two terms achieves the figural transformation of envy from a ‘diseased’ state that is dangerous to the common good into a diseased state that is dangerous to the body that experiences it. While acknowledging that the imagery of bodily corrosion is common between envy and other maladies, or emotional-states-as- maladies, such as cancer and excessive ambition, this article singled out the pathological discourse of envy. The trope of bodily corrosion speaks to concerns with elusiveness, anxiety over the power of these states for organic and social disorder, and resistance to cure or management, common to all three of them. However, in the discourse on envy it lends function to pathology. This function is to render the envier’s body diseased and diminished and by so doing to render envious desire ineffectual. In this sense the present study of envy finds the pathological discourse associated with envy predominantly figural, almost rhetorical in nature. However, this should not diminish the significance of the literal, physical ailment that envy inflicts on the early modern body.

### A Note on Further Research

Figural and literal, the discussion of envy in the early modern period is closely connected to the body. It will so remain so for most of the eighteenth century. Towards the end of the century, though, there is evidence of change. In 1799, Dr Willich in his influential treatise *Lectures on Diet*, noted that envy

[…] in general is hurtful to those only who brood over and indulge in this corrosive passion. For the world contains vast numbers, who show their envy at almost every event productive of good fortune to others, and who yet often attain a very great age ^100^.

Willich maintains here, and supports it by reference to common observation, the notion that envy is not pathological in itself but one’s attitude to it can be. The time of this observation coinciding with the emergence of obsession as a psychological category in this period as well as references to ‘monomania’ as a common diagnosis of psychological disorder ^101^, all point to a different perspective under which envy is recorded. Early-modern conceptions of envy centred on the body and suggested pathology due to the ontology of this passion. Late eighteenth-century conceptions seem to shift focus on the mind and present envy not as ‘disorder’ in itself, but, rather, as a symptom of a mental attitude that is disordered. Further research is required to determine whether there are substantial differences to warrant talk of change in the emotional register. It is hoped that future studies will take this note into consideration and explore this aspect of the history of envy further.

## Footnotes

1 For instance, from *Burton’s Anatomy of Melancholy*: ‘Envy is nothing els but Tristitia de bonis alienis, sorrow for other mens good. be it present, past, or to come: & gaudium de adversis & joy at their harmes’. Robert Burton, *The Anatomy of Melancholy: VVhat it is. Vvith all the Kindes, Causes, Symptomes, Prognostickes, and Seuerall Cures of It* (Oxford: by Iohn Lichfield and Iames Short, 1621), p. 137. ESTC no. 006193642.

2 Burton, *Anatomy of Melancholy*, p. 136.

3 Ovid, ‘The Envy of Aglauros’, in *Metamorphoses*, ed. by E.J. Kenney, trans. by A.D. Melville (Oxford: Oxford University Press, 1986), pp. 46–49, (II. 775–778).

4 ‘The Confession of Envy’, in William Langland, *Piers Plowman,* trans. by E.T. Donaldson, in *The Norton Anthology of English Literature* ed. by M.H. Abrams and others, 2 vols (New York: Norton, 1990), I, pp. 322–3 (p. 322).

5 John Skelton, ‘Philip Sparrow’, in *The Complete Poems of John Skelton*, ed. by Philip Henderson (London: J.M. Dent, 1964), pp. 60–100 (p. 86).

6 Skelton, (p. 86).

7 Edmund Spenser, *The Fairie Queene*, in *The Norton Anthology of English Literature* ed. by M.H. Abrams and others, 2 vols (New York: Norton, 1990), I, pp.622–863 (p. 669) (I.4.262–70).

8 Most sources on this aspect of envy derive from anthropological, ethnological and sociological studies. See, for example, George M. Foster, ‘The Anatomy of Envy: a Study in Symbolic Behavior’, in *Current Anthropology* 13(1972), 165–202. The manuscript sources cited here also offer examples of this social belief in the destructive power of envy. For an understanding of envy as infection in the early modern period, see also Francis Bacon, *Sylva Sylvarum: Or a Naturall History* (London: by Iohn Haviland and Aug. Mathewes, 1627), p. 251. ESTC no. 006178636.

9 Bridget K. Balint, ‘Envy in the Intellectual Discourse of the High Middle Ages’, in *The Seven Deadly Sins: From Communities to Individuals*, ed. by Richard Newhauser (Leiden, Boston: Brill, 2007), pp. 41–55 (p. 44).

10 For the use of medical metaphor in treatises on the vices, see Richard Newhauser, *The Treatise on Vices and Virtues in Latin and the Vernacular* (Turnhout: Brepols, 1993), especially page 159 on medicine as part of the conventions of the genre. See also Morton W. Bloomfield, *The Seven Deadly Sins: An Introduction to the History of a Religious Concept, With Special Reference to Medieval English Literature* (Michigan: Michigan State University Press, 1967).

11 Bloomfield, p. 221; 233 and Newhauser, *Treatise*, p. 160.

12 F.N.M Diekstra, ‘The Art of Denunciation: Medieval Moralists on Envy and Detraction’, in *In the Garden of Evil: The Vices and Culture in the Middle Ages*, ed. by Richard Newhauser (Toronto: Pontifical Institute of Mediaeval Studies, 2005), pp. 431–54.

13 Lynn S. Meskill, *Ben Jonson and Envy* (Cambridge: Cambridge University Press, 2009). For an overview of this emotion in early modern context see especially ‘An Anatomy of Envy’, pp. 42–64.

14 Judith Anderson, ‘Satanic Ethos and Envy: The Origin of Evil and Death in *Paradise Lost*’, *Milton Studies* 51(2010), 137–164.

15 Selected scholarship on the history of emotions: Barbara Rosenwein, *Emotional Communities in the Early Middle Ages* (New York: Cornell University Press, 2006); Thomas Dixon, *From Passions to Emotions: The Creation of a Secular Psychological Category* (Cambridge: Cambridge University Press, 2006); William Reddy, *The Navigation of Feeling: A Framework for the History of Emotions* (Cambridge: Cambridge University Press, 2001); Jonas Lillequist (ed.), *A History of Emotions, 1200–1800* (London: Pickering and Chatto, 2012).

16 Elena Carrera (ed.), *Emotions and Health, 1200–1700* (Leiden; Boston: Brill, 2013).

17 Fay Bound Alberti (ed.), *Medicine, Emotion and Disease, 1700–1950* (Basingstoke: Palgrave Macmillan, 2006).

18 Barbara Rosenwein, *Anger’s Past: the Social Uses of an Emotion in the Middle Ages* (Ithaca, N.Y.: Cornell University Press, 1998).

19 Elena Carrera, ‘Anger and the Mind-Body Connection in Medieval and Early Modern Medicine’, in *Emotions and Health 1200–1700*, pp. 95–146.

20 For the significance, structure, and dissemination of these health manuals see the recent work by Sandra Cavallo and Tessa Storey, *Healthy Living in Late Renaissance Italy* (Oxford: Oxford University Press, 2013), especially chapters 1, 2 and 6. On the marketplace of health manuals in England see Mary Fissell, ‘The Marketplace of Print’, in Mark Jenner and Patrick Wallis (eds), Medicine and the Market in England and its Colonies, c. 1450–1850 (Basingstoke: Palgrave, 2007), pp. 108–52.

21 See, for example, Cavallo and Storey, *Healthy Living*, especially chapter 6 ‘The Well-Tempered Man’ and pages 180–1.

22 The publication of sermons devoted to the subject of envy seems to concentrate in the late seventeenth century: Aspin, *The Envious Man’s Character*, 1684; Jonathan Blagrave, *The Nature and Mischief of Envy*, 1693; John Lambe, *A Sermon Preached Before the King*, 1695. Envy also appears in texts of obvious political nature from the middle of the century *: C. D. Vindiciae Magistratuum, of a Sober Plea for Subjection to the Present Government* (London: by Henry Hills, 1658).

23 On the Hippocratic-Galenic medical tradition, see: Owsei Temkin, *Galenism: Rise and Decline of a Medical Philosophy* (Ithaca NY: Cornell University Press, 1973); Luis García Ballester, *Galen and Galenism: Theory and Medical Practice from Antiquity to the European Renaissance* (Burlinton, VT: Ashgate, 2002). For an overview of medicine in the period studied here, see Andrew Wear, *Knowledge and Practice in English Medicine, 1550–1680* (Cambridge: Cambridge University Press, 2000); also Mary Lindemann, *Medicine and Society in Early Modern Europe*, 2nd edn (Cambridge: Cambridge University Press, 2010).

24 The view of humoural imbalance as a cause of disease can, at some points, be reductive. See also Michael Stolberg, *Experiencing Illness and the Sick Body in Early Modern Europe* (London: Palgrave Macmillan, 2011), pp. 85–9.

25 On the non-naturals see Luis García Ballester, *Galen and Galenism*, pp. 105–115. See also Lindemann, chapter 1, ‘Sickness and Health’, in *Medicine and Society*, especially pp. 13–14; 17–25.

26 Fay Bound Alberti, ‘Emotions in the Early Modern Medical Tradition’, in *Medicine, Emotion and Disease 1700–1950*, pp. 1–21 (p. 3).

27 Carrera, ‘Anger and the Mind-Body Connection’, pp. 139–143.

28 For the medieval conception of emotions in medical theories see also the work of Simo Knuuttilla: Simo Knuuttilla, ‘Medieval Theories of the Passions of the Soul’, in *Emotions and Choice from Boethius to Descartes*, ed. by Henrik Lagerlund and Mikko Yrjönsuuri (Dordrecht; London: Kluwer Academic Publishers, 2002), pp. 49–83, especially pages 56–58. Alternatively, see pages 212–218 of Simo Knuuttila, *Emotions in Ancient and Medieval Philosophy* (Oxford: Oxford University Press, 2004).

29 Pedro Gil Sotres, ‘The Regimens of Health’, in *Western Medical Thought from Antiquity to the Middle Ages*, ed. by Mirko D. Grmek (Cambridge, MA: Harvard University Press, 1998), pp. 291–318 (p. 313). This is a summarized version in the English language of Pedro Gil Sotres, ‘La higiene des las emociones’, in *Regimen sanitatis ad regem Aragononum. Arnaldi de Villanova opera medica omnia, X.1*, ed. by Luis García Ballester and Michael McVaugh (Barcelona: Fundació Noguera-Universitat de Barcelona, 1996), pp. 803–27, available from the Wellcome library.

30 Pedro Gil Sotres, ‘The Regimens of Health’ (p. 313): ‘Medieval physicians [...] reduced the number of passions from twelve to four or five, but they described in detail the somatic processes that they caused.’

31 On the lamp oil metaphor, radical moisture, and cause of death see Peter H. Niebyl, ‘Old Age, Fever, and the Lamp Metaphor’, *Journal of the History of Medicine and Allied Sciences*, XXVI (1971), 351–368; Jon Arrizabalaga, ‘Medical Causes of Death in Preindustrial Europe: Some Historiographical Considerations’, *Journal for the History of Medicine and Allied Sciences*, 54 (1999), 241–60.

32 London, British Library, MS Harley 7322, fol. 143 ^v^. The compilation includes moral and religious texts mainly in Latin. The other three similes point to senses of corrosion – ‘as rust on knife; as the worm in the tree’, and also to futility, ‘as the hul[l[ in the sea’. The poem on envy from MS Harley 7322 is included in the middle English verse miscellany by Early English Text Society, Old Series 15, p.257.-

33 John Harris, *The Divine Physician, Prescribing Rules for the Prevention, and Cure of most Diseases (*London: for George Rose, 1676), p. 53. ESTC no. 006086283.

34 Jonathan Blagrave, The *Nature and Mischief of Envy, a Sermon Preached before the Queen, Aug. 20 1693* (London: for John South, 1693), p. 14. ESTC no. 006078549

35 John Harris, *Divine Physician*, p. 52.

36 William Vaughan, *Natural and Artificial Directions for Health Derived from the Best Philosophers, as well Moderne as Auncient* (London: by Richard Bradocke, 1600), p. 51. ESTC no. 006177073. The work is the only medical work by the author going through several editions and revisions during the seventeenth-century. The work is aimed at a lay audience and contains dietetic advice, common remedies, and a section on the passions and how they affect the body.

37 London, British Library, MS Sloane 2270, fol. 5 ^r^. The text reads: ‘If thou will kiepe thee long hoale than kiepe this rule that is to say flee anger, wrath and envye give thee unto mirth and meane travail’. The British library dates the creation of the manuscript in 1530.

38 London, British Library, MS Sloane 213, fol. 112. The folio is dated around the fifteenth-century. The other manuscript containing the same sentence is: London, British Library, MS Sloane 1609, fol. 49 ^v^. The eVK, a database of early English scientific and medical texts, cites another manuscript that contains the same incipit. This is the *Ad sanitatem conservandum*, Yale Medical Library 47, fol. 103–104. The electronic research tool can be accessed in the British Library: *Scientific and Medical Writings in Old and Middle English Writings: An Electronic Reference*, compiled by Linda Voigts and Patricia Kurtz, 2000. A second edition of this database can be accessed through a link at < http://medievalacademy.org>

39 MS Sloane 2270, fol. 5 ^r^.

40 Jean Goeurot, *The Regiment of Life, Whereunto is added a Treatise of the Pestilence* (London: by T. Phayer, 1550; first publ. 1544), in *Early English Books Online* ( http://eebo.chadwyck.com [Accessed 5 June 2016]) (img 69 of 197, unpaginated). ESTC no. 006177388.

41 Virginia Langum, *Medicine and the Seven Deadly Sins in Late Medieval Literature and Culture* (New York: Palgrave, 2016), pp. 113–14.

42 C. D., *Vindiciae Magistratuum, or a Sober Plea for Subjection to the Present Government* (London: by Henry Hills, 1658), p. 21. ESTC no. 006116915.

43 David Papillon, *The Vanity of Lives and Passions of Men* (London: by Robert White, 1651), p. 404. ESTC no. 006145300.

44 Philip Moore, *The Hope of Health wherin is Conteined a Goodlie Regimente of Life* (London: by Ihon Kyngston, 1564), p. xv. ESTC no. 006181709.

45 Goeurot, *Regimen*, (img 9 of 197).

46
*The Book of Vices and Virtues: a Fourteenth Century English Translation of the Somme Le Roi of Lorens D’ Orléans*, ed. by W. Nelson Francis (London: Early English Text Society, 1942), p. 156. On the connection of melancholy to envy and anger see also ‘Appendix A: Acedia and the Humours’ in Siegfried Wenzel, *The Sin of Sloth: Acedia in Medieval Thought and Literature* (S.I.: University of North Carolina Press, 1967), pp. 191–94.

47 Lancelot Andrewes, *The Moral Law Expounded* (London: for Michael Sparke *et al*., 1642), p. 48. ESTC no. 006105825. This text compiles and publishes ‘posthumously’ notes and information from Andrewes’s theological lectures. See also *Oxford Dictionary of National Biography.*

48 Levinus Lemnius, *The Touchstone of Complexions Generallye Appliable, Expedient and Profitable for all such, as be Desirous and Careful of their Bodylye Health*, trans. by Thomas Newton (London: by Thomas Marsh, 1576), fol. 23 ^v^. ESTC no. 006203107.

49 Margaret Healy, *Fictions of Disease in Early Modern England: Bodies, Plagues and Politics* (London: Palgrave Macmillan, 2001), pp. 40–1. On this subject see also: Angus Gowland, *The Worlds of Renaissance Melancholy: Robert Burton in Context* (Cambridge: Cambridge University Press, 2006), especially pp. 33–4 ‘The nature and Status of Medical Inquiry’ and pp. 50–3 ‘Neo-Galenic Occultism’. On how to approach the heterogeneity of the early modern medical world, see also Stolberg, *Experiencing Illness*, pp. 84–5.

50 Two of the works that serve as the basis for the scheme of the four temperaments – dividing healthy people into categories according to humour and attributing separate features in each of them – ascribe the term ‘invidiosos’ to the humour black bile. These are, according to Klibansky *et al*., ‘a treatise falsely attributed to Soranus, but possibly dating from third century A.D.’ and Vindician’s *Letter to Pentadius*. Raymond Klibansky, Erwin Panofsky and Fritz Saxl, *Saturn and Melancholy: Studies in the History of Natural Philosophy, Religion and Art* (London: Nelson, 1964), p. 62.

51 Nicholas Culpeper, *The English Physitian Enlarged [...] Being an Astrologo-Physical Discourse of the Vulgar Herbs of this Nation* (London: by Peter Cole, 1653, first publ. 1652), p. 354. ESTC no. 006086018.

52 Culpeper, p. 354.

53 The connection is mostly made in the tradition of vices and virtues, but see also Virginia Langum, *Medicine and the Seven Deadly Sins* for the actual medical basis underlying this tradition. See Wenzel, ‘Appendix A’ for some examples of medieval works that link melancholy to envy and anger, p. 193.

54 Alan Chapman, ‘Astrological Medicine’, in *Health, Medicine and Mortality in the Sixteenth Century*, ed. by Charles Webster (Cambridge: Cambridge University Press, 1979), pp. 275–300; for a discussion on astrology and the meaning of the disease experience, see Michael McDonald, *Mystical Bedlam: Madness, Anxiety, and Healing in Seventeenth-Century England* (Cambridge: Cambridge University Press, 1983); also Stolberg, *Experiencing Illness*, pp. 39–41; and for information on earlier periods see Roger French, ‘Astrology in Medical Practice’, in *Practical Medicine from Salerno to the Black Death* ed. by Luis Garcia Ballester and others (Cambridge: Cambridge University Press, 1994).

55 Examples include: Robert Greene, *Planetomachia* (London: for Thomas Cadman, 1585), in *Early English Books Online* ( http://eebo.chadwyck.com/home [accessed 5 June 2016]) (img 8 of 60), ESTC no. 006177546; Thomas Tryon, *A Way to Health, Long Life and Happiness* (London: by H.C. for R. Baldwin, 1691, first publ., 1683), p. 321, ESTC no. 006126080; J. S. *The True Fortune-Teller* (London: for E. Tracy, 1698), p. 123, Wellcome Catalogue no. b10771876.

56 Klibansky *et al*., *Saturn and Melancholy*, p. 147.

57 Klibansky *et al*., pp. 137–8.

58 Klibansky *et al*., p. 146.

59 John Scott, *The Christian Life Part III: wherein the Great Duties of Justice, Mercy, and Mortification are fully Explained and Inforced* (London: for Walter Kettilby, 1696), p. 309. ESTC no. 006125320.

60 Robert Dixon, *Canidia, or The Witches, a Rhapsody in five parts* (London: by S. Roycroft for Robert Clavell, 1683), in *Early English Books Online* < http://eebo.chadwyck.com/home> [accessed 5 June 2016] (img 160 of 304). ESTC no. 006077608. The work is a verse satire on social ills by a religious author.

61 On the nature of black bile and Galenism see also Mark Grant, ‘On Black Bile’, in *Galen on Food and Diet* (London and New York: Routledge, 2000), pp. 19–36. On black bile and its connection to the three types of melancholia see also’Black Bile and Melancholia: Avicenna’, in Jennifer Radden (ed.), *The Nature of Melancholy from Aristotle to Kristeva* (Oxford: Oxford University Press, 2000), pp. 75–8.

62 Erin Sullivan, *Beyond Melancholy: Sadness and Selfhood in Renaissance England* (Oxford: Oxford University Press, 2016), especially pages 98–108.

63 Rudolph Siegel, *Galen’s System of Physiology and Medicine: an Analysis of his Doctrines and Observations on Bloodflow, Respiration, Humors and Internal Diseases* (Basel: Karger, 1968), p. 258.

64 Siegel, p. 258.

65 Lemnius, *Touchstone*, fol. 69 ^r^.

66 An overview of the processes of digestion and assimilation can be found in Ken Albala, *Eating Right in the Renaissance* (Berkeley and Los Angeles: University of California Press, 2002), pp. 54–63.

67 Albala, p. 54.

68 Ovid, *Metamorphoses*, p. 47, (II. 769).

69 Spenser, *Fairie Queene*, p. 669, (I.4. 266).

70 Langland, *Piers Plowman*, p.323, lines 121–25. For a discussion of the physicality of Langland’s envy see also Langum, p. 113; Rosanne Gasse, ‘The Practice of Medicine in *Piers Plowman*’, *The Chaucer Review*, 39.2(2004), 177–97.

71 Ovid, *Metamorphoses*, pp. 47–8, (II. 777–8).

72 Langland, *Piers Plowman*, p. 322, lines 83 and 86.

73 Spenser, *Fairie Queene*, p.669, (I.4. 263–64).

74 Stolberg, p. 123.

75 Albala, p. 54.

76 Thomas Adams, *Diseases of the Soule: A Discourse Divine, Moral and Physical* (London: by George Purslowe, 1616), p. 18. ESTC no. 006172069. The writer is obscure but the text is among a long tradition of texts that treat illness as both spiritual and physical.

77 ‘Envy’, in *The Rich Cabinet furnished with a Variety of Excellent Discriptions* (London: by Iohn Beale, 1616), pp. 41–46 (p. 44).

78 John Scott, *The Christian Life Part III*, p. 309.

79 The anecdote appears as a note in a translation of Luigi Cornaro’s (1467–1565) *Sure and Certain Methods of Attaining a Long and Healthy Life* (Philadelphia: Reprinted for the Rev. M. L. Weems, 1793; first publ. 1558), pp. 36–37, p 36. ESTC no. 006457396. It is difficult to ascertain the exact date and author of this anecdote on envy as Cornaro’s work went through multiple editions from its original conception to the nineteenth century, with differences in the text. According to William Walker, the 1793 American edition is based on the 1737 translation, ‘Luigi Cornaro, a Renaissance Writer on Personal Hygiene’, *Bulletin of the History of Medicine* 28 (1954), 525–34 (p. 531).

80 See, for example, the discussion of envy by Aaron Ben-Ze’ ev, ‘Aristotle on Emotions Towards the Fortune of Others’ , in *Envy, Spite and Jealousy: The Rivalrous Emotions in Ancient Greece*, ed. by David Konstan and Keith Rutter (Edinburgh: Edinburgh University Press, 2003), pp. 99–121 (p. 108).

81 Richard Allestree, Sermon XXI. :‘The Light of the Body is the Eye’, in *Forty Sermons whereof Twenty One are now Firstly Publish’d, the greatest part Preach’d before the King on Solemn Occasions* (Oxford and London: for R. Scott *et al*., 1684), pp. 284–95 (p. 286). ESTC no. 006146088.

82 Everard Maynwaringe, *Tutela Sanitatis sive Vita Protracta: The Protection of Long Life, and Detection of its Brevity, from Diaetic Causes and Common Customs* (London: by Peter Lillicrap, 1664), p. 57. ESTC no. 006120566.

83 Specifically, these are: *The Protection of Long Life, and Detection of its Brevity* (London: by Peter Lillicrap, 1664); *The Preservation of Health and Prolongation of Life Proposed and Proved* (London: by J.D., 1669); and T *he Method and Means of Enjoying Health, Vigour, and Long Life* (London: by J.M, 1683).

84 Maynwaringe, *Method,* p. 202; *Preservation*, p. 153.

85 Alanna Skuse, *Constructions of Cancer in Early Modern England: Ravenous Natures* (Palgrave Macmillan, 2015) Open Access.

86 On the failure of the Helmontians, see Wear, Knowledge and Practice, pp. 399–433. See also Lindemann, pp. 102–3.

87 Francis Bacon, ‘Envy’, in *Francis Bacon; The Essays*, ed. by John Pitcher (London: Penguin, 1985), p. 28.

88 Ibid.

89 Consumption is defined closely to the sense of atrophy as a consequence of disease and also differentiated as disease on its own. In the discourse on envy is used in the former sense, see for example James Primrose, *Popular Errours.Or the Errours of People in Physick* (London: by W, Wilson, 1651), p. 97.

90 Information on this section derives from Skuse, *Constructions of Cancer*,

91 See William Casey King, *Ambition, a History: from Vice to Virtue* (New Haven: Yale University Press, 2013).

92 Adams, *Disease*s, p. 40.

93 Burton, *Anatomy of Melancholy*, p. 150.

94 John Lambe’s sermon on envy (for Walter Kettilby, 1695), noted that there should be no reason for envy as ‘the goods of the world, of body, mind, and fortune, are dispenc’d in measures and degrees amongst us all’. (p. 18).

95 Skuse, *Constructions*, p. 90.

96 The term ‘corrosion’ - and its derivatives, ‘corroding’ and ‘corrosive’ - as a term of physiology appears mainly in surgical treatises and is also associated with cancer. The definitions used here appear in Anon, *A Physical Dictionary* (London:by G. D. for John Garfield, 1657), p. 33; William Bullein, *Bullein’s Bulwarke of Defence against all Sicknesse* (London: by Thomas Marshe, 1579), index.

97 Adams. *Diseases*, p. 42.

98 Adams, p. 41,

99 On the significance of the process of concoction in the matter of cure see Hannah Newton, ‘“Nature Concocts & Expels”: The Agents and Processes of Recovery from Disease in Early Modern England’, Social History of Medicine 28.3(2015), 465–86 (pp. 475–6).

100 A. F. M. Willich, *Lectures on Diet and Regimen: Being a Systematic Inquiry into the most Rational Means of Preserving Health and Prolonging Life* (London: for T.N. Longman and O. Rees, 1799), p. 592. ESTC no. 006364625.

101 Lennard J. Davis, *Obsession: a History* (Chicago and London: University of Chicago Press, 2008), especillay chapter 2. ‘The emergence of obsession’ and 3. ‘Specialization as Monomania’.

