# Supplementary material for: Envy's pathology: Historical contexts
Source: Wellcome Open Res. 2017 May 23;2:3. Originally published 2017 Jan 9. [Version 2] doi: 10.12688/wellcomeopenres.10415.2 (PMC5500894; doi:10.12688/wellcomeopenres.10415.2)
Supplement: Peer review history

# Peer review: Referee response for version 2

**DOI:** 10.21956/wellcomeopenres.12607.r23778

**Published:** 2017-06-27

**Authors:** Javier Moscoso

**Affiliations:** 1 Institute History, Centre for Humanities and Social Sciences, Spanish National Research Council, Madrid, Spain

I believe the paper has gained very much in terms of clarity and scope. I like in particular the way in which the first sections have been re-written, using new secondary sources, but also adding much clarity to the core of the discussion. I am also very much satisfied regarding the introduction of cancer and ambition in comparison to envy. All in all, I believe this a very informative and thought-provoking paper about the phsyiological understanding of envy in Early Modern Culture. I am glad that Wellcome Open Research Team has given me the opportunity to participate in this endevour, and grateful to Lina Minou for having taking so seriously my previous comments.

I have read this submission. I believe that I have an appropriate level of expertise to confirm that it is of an acceptable scientific standard.

# Peer review: Referee response for version 2

**DOI:** 10.21956/wellcomeopenres.12607.r22963

**Published:** 2017-06-02

**Authors:** Hannah Newton

**Affiliations:** 1 Department of History, University of Reading, Reading, UK

I am very happy with the author's revisions, which I think have significantly strengthened the article. In particular, the adjustments to the structure, with the addition of 'background' section, and its separation from the 'methods' part, has served to clarify the opening sections. The extra information about the sources, and their potential limitations, gives the reader a fuller sense of the research methods. The overarching argument - about the pernicious physicality of envy - is now more powerfully and persuasively conveyed. It is also great to see that the author has engaged with some additional secondary works, integrating their respective insights seamlessly into the text. Finally, I think the question of change over time has now been addressed much more subtly and convincingly. My thanks to Lina Minou for her revisions, and for giving me the opportunity to read her very interesting and insightful piece.

I have read this submission. I believe that I have an appropriate level of expertise to confirm that it is of an acceptable scientific standard.

# Peer review: Referee response for version 1

**DOI:** 10.21956/wellcomeopenres.11222.r19111

**Published:** 2017-01-23

**Authors:** Javier Moscoso

**Affiliations:** 1 Institute History, Centre for Humanities and Social Sciences, Spanish National Research Council, Madrid, Spain

This is a very interesting and informative paper about the physiological symptoms of envy, as they were described in, mainly, English medical treatises of the early modern period. The paper claims that the modern discourse on envy was always pathological, essentially due to the way in which envy was interpreted and understood within a humoral theory of disease. The paper sees a historical change in the physiological, but also a evaluative, understanding of envy, corresponding with the transition from humoralism to a mechanical conception of disease; a shift that the author associates with the coming about of a new “paradigm” in the 18th century. The paper focuses on the physiological effects that envy has on the envier, but also touches on some other wider issues, like the social consideration of envy as a dangerous social passion that connoted social strife and disorder.

 To begin with, the paper does not include any kind of geographical restrictions in its methodological assumptions or in its conclusions. Though the bulk of the text is based on English sources or English translations, the author seems to discuss emotional shifts in an ideal territory that could perhaps be identified with Europe, but not necessarily so. This should be clarified, for two important reasons.

 First of all, the tendency to identify English history with the whole of European, or Global History, does not seem to agree with current historiographical trends, including post-colonial or global history. Though these kinds of restrictions should always be clear, it is even more so when dealing with the history of the emotions, a field in which geographical or linguistic differences may reflect important cultural distinctions, as cultural anthropology but also cultural history has come to show very often. From a cultural point of view, the expression, but also the physiological experience of emotions may vary depending on the cultural forms in which those very emotions, or passions, are discussed, expressed, or repressed. Still more important for the general thesis of the paper, it might well be that the shift from a physiological experience of envy based on humoralism to a more enlightened model, does not simply apply to many other European or American cultural regions. In Spain and Italy, but also in France, discussion on passions was very often based on humoral theory well up to the mid-19 ^th^ century. A simple look at early 19 ^th^ century French medical thesis on passions will suffice to make us gain a different picture regarding the continuity or discontinuity of humoral theory of passions beyond the Enlightenment .

 Secondly, the discussion on envy touches on many other passions, including anger or sorrow. In some occasions, the paper seems to imply that “envy” is a kind of entity that could be divided into “its core elements” (p. 6). The connection between envy and melancholy, of which the papers offers plenty of examples, seems to rely on this kind of relation. On the one hand, the melancholic “was physiological inclined to be envious” (p. 7), whereas on the other, the envious was also described with melancholic features “All the diseases that converge on the black bile are part of the language of envy” (p. 9). This association between the physiology of passions and their moral or philosophical characterization remains the most intriguing and exciting part of the paper. The author might consider, however, to drop the mechanical understanding of history, based on “medical paradigms”, and consider instead, the analysis of the signs of envy inscribed in the body or verbalized by the use of the language. This might offer a much better understanding of the connection between envy and similar passions and conditions: melancholy, of course, but also different forms of ambition, including social and economic ambition. From this point of view, the discussion about the fate of envy during the European Enlightenment would take a quite different approach in terms of continuity or discontinuity. By focusing on the history of the physiological signs, independent of their social or conceptual framing, the paper could also introduce new considerations regarding the history of cancer, and some other incurable diseases, described very often in terms of a corrosion of the body caused by sadness, envy, ambition of different kinds, and so on.

 In brief, this is a very interesting paper based on first class research. It could be indexed with two important modifications. First, the author should clarify its geographical and cultural scope. The general thesis that the paper defends does not simply correspond with evidences from other European sources and languages. Secondly, the historiographical model that informs the text, based on a mechanical substitution of medical paradigms could be informed by a discussion on the transformation of (physiological) signs into (medical or moral) symptoms. The history of physiological signs will also provide some interesting connections with the history of some other conditions, including the experience of cancer in early modern Europe.

 Some other minor, considerations:

 Note 25: please check Pedro Gil Sotres, La higiene de las emociones (instead of des emociones). In the same note, Luis García Ballester (and no Garcia)

I have read this submission. I believe that I have an appropriate level of expertise to confirm that it is of an acceptable scientific standard, however I have significant reservations, as outlined above.

# Response

**Published:** 2017-01-25

**Authors:** Lina Minou

**Affiliations:** Independent researcher , UK

Thank you very much for your review. You make some very fair and significant points and I am grateful for your insights. I will work to address these points in the forthcoming revised version of this article.

# Peer review: Referee response for version 1

**DOI:** 10.21956/wellcomeopenres.11222.r19112

**Published:** 2017-01-16

**Authors:** Hannah Newton

**Affiliations:** 1 Department of History, University of Reading, Reading, UK

This is a richly textured analysis of the perceived effects of the passion of envy on human health in England between the sixteenth and eighteenth centuries. The author argues convincingly that this emotion was regarded – both by medics and spiritual writers - as unequivocally pernicious to the body, unlike some other related passions, such as anger. Associated with the dry, cold humour, ‘melancholy’, it hindered the vital process of digestion, and ultimately could kill by depriving the body of vital heat. The article makes a valuable contribution to the growing historiography on ‘embodied emotion’, and the links between mental and physical health in early modern culture. There are, however, a number of ways in which the article could be developed and strengthened, outlined below.

*Opening section*

 I wondered if the opening could be made slightly more imaginative, perhaps with use of one of the colourful quotations in the section on the language of envy. The personification of envy, as a leathery-eyed, creaky-boned individual is very striking, and might make a nice way in. In the Introduction, it would also be helpful to be told how early modern people defined emotion (or 'passion' to use their terminology), together with envy (and how it differed – if it did – from jealousy); at the moment, envy is defined on p.2, but the definition has been paraphrased - it would be more powerful to have a contemporary quotation here, and for it to be moved up to the first page.

*Historiographical contribution and engagement*

 The article displays a sound knowledge of the literature of emotion, both of what has been written specifically on envy, and on the broader scholarship of early modern health and the passions. Several additional important monographs, however, could be brought in where relevant, including:

- Sandra Cavallo and Tessa Storey, *Healthy Living in Late Reniassance Italy *(2013): contains a chapter on emotions and health.

- Erin Sullivan, *Beyond Melancholy: Sadness and Selfhood in Renaissance England *(Oxford, 2016); this wonderful book is very relevant to your interest in the humour melancholy, and its effects on the body as well as the mind.
- Alanna Skuse, *Constructions of Cancer in Early Modern England: Ravenous Natures *(Basingstoke, 2015): discusses the imagery of cancer in a way that resonates nicely with your analysis of the metaphors of ‘worms’ and ‘cankers’ in descriptions of envy.

When thinking about the ‘gap’ in the literature, and how this article advances knowledge, it occurred to me that one thing it does well, is reveal the interrelationships between individual passions (envy and anger, envy and sadness, etc); as far as I know not a huge amount of work has been carried out on this subject, and it is one which I think you could highlight more explicitly.

*Methodology and structure*

 In the Methods and Sources section, it is useful to hear how the sources have been selected, but I think a bit more needs to be said about the potential limitations of the sources and search methods. What are the pitfalls to using key word searches, and how have you got around them? For instance, were there other words, which have fallen out of use today, that may have been used in place of the word ‘envy’? How far do the chosen primary texts reflect the attitudes of most people in society? Is it ever possible to access the perceptions of poorer people, who left few records? I expect some popular ballads could be found on envy, which may provide some insights, albeit indirect ones.

 In this section, it would also be useful to be given a little more information about the purposes, authors, and nature of the various primary sources, including the spiritual/philosophical texts which at the moment are not defined. Indeed, whenever a quotation or example is given, it would be worth giving a word or two of biographical information about the author (even if just their dates). Later in the article (p.8), when discussing Lemnius’ treatise, an interesting point is made about how medical texts tend to mention envy less frequently than religious/philosophical texts, owing to the fact that this emotion did not illustrate the point they wanted to make about the heart and spirits; the heterogeneity of texts in the period is also highlighted here. I felt that this observation needed to be moved to the Methods section, as it justifies why you’ve used philosophical/religious texts as well as medical ones.

 My other comment about the methodology section is that at the moment it is interspersed with quite detailed explanations of medical theory, e.g. the non-naturals. It might be clearer for the reader if this information could be put into a separate introductory section, perhaps headed ‘Background on Emotion and Health’. The passage explaining the concepts of the spirits and natural heat (currently on p.5) could be inserted here too. The rest of the article’s structure seems effective.

*Argument and scope*

 I think the argument that the effects of envy were physical as well as symbolic, is convincing, especially in relation to the loss of nutrition and natural heat. What I felt could be clarified slightly is whether the disease-specific metaphors (comparing envy to plague, leprosy, etc) were meant literally. Did envy actually make people more susceptible to contracting these diseases? If so, how did it do this? Perhaps such phrases were meant both metaphorically and literally. Could envy also cause forms of mental illness, like love sickness and mania?

 Continuing the subject of metaphors, on p.1 it is stated that medical metaphors were also applied to the spiritual impact of envy – this passion was a disease of the soul, a deadly vice, etc; the implication is that the whole idea of ‘spiritual health’ is a metaphor. From a secular viewpoint, I can see why this language seems metaphorical, but I don’t think that early modern people would have viewed it in this way. They believed that human beings consisted of two parts – the body and soul – and both were capable of health and disease. Thus, when the soul was said to be sick of sin (of which envy was one), they did not mean this metaphorically, but literally. I think this could be clarified in the text.

 When discussing the ‘evil eye’ of envy, there might be scope to bring in the sense of sight. Why was this sense associated with envy? Do different passions connect with different senses?

 Finally, a comment on the final section on change over time: I was convinced by the argument that envy was no longer regarded as pernicious to everyone in society (i.e. it was only dangerous to those who ‘indulged’ in it etc). However, the statements about the radical transformation in medical theory and practice perhaps over-state the extent of change, and could be softened. Various historians have shown that the basic belief in the humours (or ‘morbid matter’), and the need for rebalancing and purging, persisted into the eighteenth century, despite the ‘discoveries’ of the likes of Harvey and Newton. Although Boerhaave’s new emphasis on the role of motion and stagnation might seem new, it did in fact bear some resemblance to the older humoral idea that disease could be caused by the obstruction or putrefaction of the spirits/humours, and that health depended on a continual flowing. Many of the traditional humoral remedies aimed at unblocking obstructions, and the reason that emotions like cheerfulness were good for health was because they encouraged the lively movement of the spirits about the body (see Cavallo & Storey’s chapter).

 In order to incorporate some of the above ideas within the word limit, I think there’s potential for a bit of pruning of words, especially in the introductory section.

*Minor comments *

- Top of p.3: the sentence ‘potent image of body-related imagery’ – use of ‘image’ twice in one sentence. Could one of them be removed?
- p.3 ‘concept of study relates to the concept of sin’-  do you mean ‘concept under study’?
- p.3. the word ‘surge’ is used twice in 2 sentence close together.
- p.3 missing comma after Fay Bound Alberti.
- p.3. ‘late seventeenth’ needs ‘century’ adding I think.
- p.4: missing comma: ‘with the exception of a small number of manuscripts the texts used here are usually print sources drawing, directly or indirectly, from the principles of the Hippocratic-Galenic tradition’. The comma is needed after ‘manuscripts’.
- p.5 first column: missing comma after ‘opposite effect’.
- p.5. I think the tense needs to be changed: ‘By contrast, hectic fever has different connotations. It is a very specific pathological condition that is the result…’ – should this be ‘hectic fever had different connotations. It was a very specific pathological…’?
- p.5. The sentence, ‘This tendency towards the ultimately harmful is usual to envy’ – could be rephrased for clarity- e.g. ‘This tendency to view envy as potentially fatal is usual’.
- p.5. The quote from the Harley collection needs a little bit more contextualising I think.
- p.6. The sentence, ‘sorrow is not adequate to describe the experience of envy’ – might need re-phrasing for clarity – e.g. ‘the experience of sorrow was not the same as that of envy’.
- at the end of the section on language, the relationship between anger and envy is introduced. This could be moved to the start of the next section (on Anger and Envy).
- p.7: ‘Except for movement of spirits, anger is also connected to heat’. This should be rephrased, as the meaning isn’t quite clear. Do you mean that besides the different movement of spirits, anger was similar to envy in terms of its association with heat?
- p.11. ‘the idea of humoural imbalance as [the] cause of disease’. (missing ‘the’).
- p.11, in the section on Helmontians: as Helmontians supposedly didn’t believe in the humours or the qualities they represented, it might seem strange that they could associatae envy with dryness. Maybe comment on this and offer a possible reason. Was it simply too ingrained in the culture of the time not to use?

I have read this submission. I believe that I have an appropriate level of expertise to confirm that it is of an acceptable scientific standard, however I have significant reservations, as outlined above.

# Response

**Published:** 2017-01-25

**Authors:** Lina Minou

**Affiliations:** Independent researcher , UK

Thank you very much for your review. I will work to address your concerns, alongside the points expressed in the second referee report, in a revised version of this work. Thank you also for taking the time to be detailed in your review and for suggesting relevant sources.
